# Nonlinear Spatial Integration Underlies the Diversity of Retinal Ganglion Cell Responses to Natural Images

**DOI:** 10.1523/JNEUROSCI.3075-20.2021

**Published:** 2021-04-14

**Authors:** Dimokratis Karamanlis, Tim Gollisch

**Affiliations:** ^1^Department of Ophthalmology, University Medical Center Göttingen, Göttingen, 37073, Germany; ^2^Bernstein Center for Computational Neuroscience Göttingen, Göttingen, 37073, Germany; ^3^International Max Planck Research School for Neurosciences, Göttingen, 37077, Germany

**Keywords:** natural visual stimuli, nonlinear spatial integration, receptive field, retinal ganglion cell

## Abstract

How neurons encode natural stimuli is a fundamental question for sensory neuroscience. In the early visual system, standard encoding models assume that neurons linearly filter incoming stimuli through their receptive fields, but artificial stimuli, such as contrast-reversing gratings, often reveal nonlinear spatial processing. We investigated to what extent such nonlinear processing is relevant for the encoding of natural images in retinal ganglion cells in mice of either sex. We found that standard linear receptive field models yielded good predictions of responses to flashed natural images for a subset of cells but failed to capture the spiking activity for many others. Cells with poor model performance displayed pronounced sensitivity to fine spatial contrast and local signal rectification as the dominant nonlinearity. By contrast, sensitivity to high-frequency contrast-reversing gratings, a classical test for nonlinear spatial integration, was not a good predictor of model performance and thus did not capture the variability of nonlinear spatial integration under natural images. In addition, we also observed a class of nonlinear ganglion cells with inverse tuning for spatial contrast, responding more strongly to spatially homogeneous than to spatially structured stimuli. These findings highlight the diversity of receptive field nonlinearities as a crucial component for understanding early sensory encoding in the context of natural stimuli.

**SIGNIFICANCE STATEMENT** Experiments with artificial visual stimuli have revealed that many types of retinal ganglion cells pool spatial input signals nonlinearly. However, it is still unclear how relevant this nonlinear spatial integration is when the input signals are natural images. Here we analyze retinal responses to natural scenes in large populations of mouse ganglion cells. We show that nonlinear spatial integration strongly influences responses to natural images for some ganglion cells, but not for others. Cells with nonlinear spatial integration were sensitive to spatial structure inside their receptive fields, and a small group of cells displayed a surprising sensitivity to spatially homogeneous stimuli. Traditional analyses with contrast-reversing gratings did not predict this variability of nonlinear spatial integration under natural images.

## Abstract (precis)

How neurons encode natural stimuli is a fundamental question for sensory neuroscience. In the early visual system, standard encoding models assume that neurons linearly filter incoming stimuli through their receptive fields, but artificial stimuli, such as contrast-reversing gratings, often reveal nonlinear spatial processing. We investigated to what extent such nonlinear processing is relevant for the encoding of natural images in retinal ganglion cells in mice of either sex.

## Introduction

The natural visual world is communicated to the brain through an array of functionally distinct parallel channels that originate in the retina (Roska and Meister, 2014; Baden et al., 2016). A classical view of retinal function advocates that the retinal output channels, represented by types of retinal ganglion cells (RGCs), serve as linear filters for natural visual inputs (Atick and Redlich, 1990; Shapley, 2009). Recordings under artificial visual stimuli, such as contrast-reversing gratings (Enroth-Cugell and Robson, 1966; Demb et al., 1999; Petrusca et al., 2007; Krieger et al., 2017) or finely structured white noise (Freeman et al., 2015; Liu et al., 2017), however, have shown that several ganglion cell types have spatially nonlinear receptive fields (RFs). The nonlinearities arise in the RF center from the nonlinear integration of excitatory signals, which originate from presynaptic bipolar cells (Demb et al., 2001; Borghuis et al., 2013; Turner and Rieke, 2016). Furthermore, nonlinear RFs are proposed in circuit models of retinal computations that are thought to occur during natural vision (Gollisch and Meister, 2010), such as the distinction of object from background motion (Ölveczky et al., 2003; Baccus et al., 2008; Zhang et al., 2012).

Together, these findings raise the question to what extent nonlinear RFs of different ganglion cell types play a role in natural vision. On the one hand, the spatial structure of natural stimuli is not as pronounced and rich in high spatial frequencies as in typical artificial stimuli used to detect nonlinear RFs because the light intensities of nearby regions in natural images are correlated (Burton and Moorhead, 1987). This leads to extensive areas of nearly homogeneous illumination, for which RF nonlinearities may play no role. On the other hand, object boundaries can induce pronounced changes of stimulus intensity over short distances (Turiel and Parga, 2000), and textures or illumination gradients may provide further structure within individual RFs. Despite the importance of evaluating stimulus encoding models under natural stimuli (Carandini et al., 2005; Felsen and Dan, 2005), only few studies have focused on whether the linear RF provides a good abstraction of RGCs for stimuli with natural spatial structure, and reported findings are mixed. Some studies support that linear RFs suffice to describe natural stimulus encoding in mouse and primate retina (Nirenberg and Pandarinath, 2012; Bomash et al., 2013), whereas others indicate that linear RFs may fail to predict natural scene responses in mammalian (Cao et al., 2011; Freeman et al., 2015; Heitman et al., 2016; Turner and Rieke, 2016; Shah et al., 2020) and salamander retinas (Liu et al., 2017; McIntosh et al., 2017).

In this work, we establish a connection of spatial RF nonlinearities to the encoding of natural images in RGCs. We do so in the mouse retina, in which spatial integration, as measured with artificial stimuli, appears to display a broad scope (Carcieri et al., 2003), with spatially linear (Krieger et al., 2017; Johnson et al., 2018) as well as strongly nonlinear cells (Zhang et al., 2012; Jacoby and Schwartz, 2017; Mani and Schwartz, 2017). We first show that linear RF models successfully predict responses to natural images for some ganglion cells and substantially fail for others. We then connect model failure to the characteristics of spatial nonlinearities in the RF center and analyze these under different stimulus layouts and for specific functional cell types.

## Materials and Methods

### 

#### 

##### Experimental design and statistical analysis

We used 13 retina pieces from 9 adult WT mice of either sex (6 C57BL/6J and 3 C57BL/6N; 7 male and 2 female), mostly between 8 and 12 weeks old (except for one 18- and one 26-week-old). All mice were housed in a 12 h light/dark cycle. Experimental procedures were in accordance with national and institutional guidelines and approved by the institutional animal care committee of the University Medical Center Göttingen, Germany. No statistical methods were used to predetermine sample size. Statistical tests and associated information (e.g., *p* values) are noted where appropriate in the text. For all statistical procedures, we used default MATLAB2019b functions.

##### Tissue preparation and electrophysiology

Mice were dark-adapted for at least 1 h before eye enucleation. After the animal had been killed, both eyes were removed and immersed in oxygenated (95% O_2_-5% CO_2_) Ames' medium (Sigma Millipore), supplemented with 22 mm NaHCO_3_ (Merck Millipore) and 6 mm D-glucose (Carl Roth). We cut the globes along the ora serrata, removing the cornea, lens, and vitreous humor. In some experiments, the resulting eyecups were cut in half to allow two separate recordings. Before the start of each recording, we isolated retina pieces from the eyecups. We placed the pieces ganglion cell-side-down on planar multielectrode arrays (Multichannel Systems; 252 electrodes; 30 μm diameter, either 100 or 200 μm minimal electrode distance) with the help of a semipermeable membrane, stretched across a circular plastic holder (removed before the recording). The arrays were coated with poly-D-lysine (Merck Millipore). Throughout the recording, retinal pieces were continuously superfused with the oxygenated Ames solution flowing at ∼250 ml/h. The bath solution was heated to a constant temperature of 34°C-35°C via an inline heater in the perfusion line and a heating element below the array. Dissection and mounting were performed under infrared light on a stereo-microscope equipped with night-vision goggles.

Extracellular voltage signals were amplified, bandpass filtered between 300 Hz and 5 kHz, and digitized at 10 kHz sampling rate. Spikes were detected by threshold crossings (4 SDs of the voltage trace), and spike waveforms were sorted offline into units with a custom-made IgorPro (WaveMetrics) routine based on Gaussian mixture models (Pouzat et al., 2002). We curated the routine's output and selected only well-separated units with clear refractory periods. Duplicate units were identified by temporal cross-correlations and removed. Finally, only units with stable electrical images (Litke et al., 2004) throughout the recording were considered for further analysis.

##### Visual stimulation

Visual stimuli were generated and controlled through custom-made software, based on Visual C++ and OpenGL. Different stimuli were presented sequentially to the retina through a gamma-corrected monochromatic white OLED monitor (eMagin) with 800 × 600 square pixels and 60 Hz refresh rate. The monitor image was projected through a telecentric lens (Edmund Optics) onto the photoreceptor layer of the retina, and each pixel's side measured 7.5 μm on the retina. All stimuli were presented on a background of low photopic light levels (2.5 or 3.5 mW/m^2^, corresponding to 1500 or 1900 R*/rod/s), and their mean intensity was always equal to the background. We fine-tuned the focus of stimuli on the photoreceptor layer before the start of each experiment by visual monitoring through a light microscope and by inspection of spiking responses to contrast-reversing gratings with a bar width of 30 μm.

##### Linear RF identification

To estimate the RF of each cell, we used a spatiotemporal binary white-noise stimulus (100% contrast) consisting of a checkerboard layout with flickering squares (60 μm side). The update rate was either 30 or 60 Hz in different experiments. We measured the spatiotemporal RF by calculating the spike-triggered average (STA) over a 500 ms time window (Chichilnisky, 2001) and fitted a parametric model to the RF (Chichilnisky and Kalmar, 2002). The model was spatiotemporally separable and comprised a product of a spatial ($k_{S}(x)$) and a temporal component ($k_{T}(t)$).

The spatial component was modeled as a difference of Gaussians as follows:

$$k_{S}\left(x\right)=N\left(x;\mu ,\Sigma \right)-A_{S}N\left(x;\mu ,k^{2}\Sigma \right)$$

where $N\left(x;\mu ,\Sigma \right)=e^{-\frac{1}{2}{\left(x-\mu \right)}^{T}\Sigma^{-1}(x-\mu )}$ is a two-dimensional Gaussian function with mean μ and covariance matrix Σ (describing the RF center's coordinates and shape), $A_{s}\in [0\text{,}1]$ captures the RF surround strength relative to the RF center, and k≥1 is a scaling factor for the surround's extent.

The temporal component was modeled as a difference of two low-pass filters as follows:

$$k_{T}\left(t\right)=p_{1}{\left(\frac{t}{\tau_{1}}e^{-\frac{t}{\tau_{1}}+1}\right)}^{n}-p_{2}{\left(\frac{t}{\tau_{2}}e^{-\frac{t}{\tau_{2}}+1}\right)}^{n}$$

with t>0 indicating the time before the spike and $p_{1}>0$, $p_{2}>0$, $\tau_{1}>0$, $\tau_{2}>0$, n>0 being free parameters.

We fitted the full parametric model ($k_{S}(x)\cdot k_{T}(t)$) to the STA by minimizing the mean squared error using constrained nonlinear optimization. To get reasonable initial conditions, we first separately fitted the spatial component to the STA frame at which the element with the largest absolute value occurred and the temporal component to the time course of the same element. If the element was negative, the sign of the STA frame was inverted before the spatial component fit. We then seeded the obtained values of spatial and temporal fits as the initial parameters for the full spatiotemporal fit. The chosen initialization procedure is consistent with the positive center peak of $k_{S}$.

The diameter of the RF center was defined as the diameter of a circle with the same area as the 2σ (elliptical) boundary of the Gaussian center profile (Baden et al., 2016). We also used the 2σ boundary for all RF center visualizations.

##### Natural image response predictions with a linear-nonlinear (LN) model

We selected natural images as stimuli from three sources: the van Hateren Natural Image Dataset (van Hateren and van der Schaaf, 1998), the McGill Calibrated Color Image Database (Olmos and Kingdom, 2004), and the Berkeley Segmentation Dataset (Arbeláez et al., 2011). The central square region of each image was resized to 512 × 512 pixels (400 × 400 pixels in a few experiments) by cropping (van Hateren and McGill images) or cropping and upsampling with nearest neighbor interpolation (Berkeley images). All color images (McGill and Berkeley databases) were converted to grayscale by weighted averaging over the color channels (Liu et al., 2017). We normalized the mean and SD of the pixel values for each image by appropriately shifting and scaling the values so that the mean pixel intensity was equal to the background and the SD was 40% of the mean intensity. Pixel values that then deviated from the mean by >100% in either direction were clipped to ensure that the maximal pixel values were within the physically available range of the display. Finally, all images were encoded at 8-bit color depth to match the range of our OLED monitor. The images were presented on top of a uniform gray background and centered on the multielectrode array, covering a region of 3.84 × 3.84 mm^2^ on the retina (3 × 3 mm^2^ for 400 × 400 pixels).

In every experiment, we used 300 natural images (100 from each database), except for one (200 images in total). Images were presented individually for 200 ms each, with an 800 ms interstimulus interval of homogeneous background illumination. We collected 10 trials for each image by consecutively presenting 10 different pseudo-randomly permuted sequences of all images. For each cell, we measured the response as the trial-averaged number of spikes over a 250 ms window following stimulus onset.

To compare a cell's responses to model predictions, we constructed an LN model (Chichilnisky, 2001), which generates average spike count responses $R_{m}\geq 0$ to natural image stimuli $s_{m}$: $R_{m}=f(k^{T}\cdot s_{m})$, where the vector k is a linear spatial filter, f is a nonlinear function, and m denotes the image index. For the analyses, all natural image stimuli were spatially clipped to the smallest square that could fit the 4σ boundary of the RF center, and their pixel intensity values were transformed to Weber contrast values, which constitute the elements of $s_{m}$. For the linear filter (k), we used the parametric spatial RF component ($k_{S}$) estimated from white noise, sampled at the center point of each pixel of the clipped natural image. The linear filter was normalized to a sum of unity of the absolute values of its elements.

In initial analyses, we also tried using the pixelwise spatial profile obtained from the reverse-correlation analysis as a spatial filter of the model. Overall, response predictions and model performances were similar to the ones under the parametric fit, yet often more noisy, owing to noise in the pixelwise filter estimate. All further analyses were therefore based on the parametric fit of the spatial filter.

Linear predictions (g) were estimated from the inner product of stimuli and the linear filter: $g_{m}=k^{T}\cdot s_{m}$. Because the linear filter is composed of mainly positive values, the sign of the linear prediction reflects the net contrast in the spatial RF. For the nonlinear part of the LN model (f), we used a bi-logistic nonlinearity of the following form:

$$f\left(g\right)=b+\frac{M_{ON}-b}{1+e^{-r_{ON}\left(g-g_{ON}\right)}}+\frac{M_{OFF}-b}{1+e^{r_{OFF}\left(g-g_{OFF}\right)}}$$

where b≥0, $M_{ON}\geq b$, $M_{OFF}\geq b$, $r_{ON},\geq 0$, $r_{OFF}\geq 0$, and $g_{ON}$, $g_{OFF}$ were free parameters that were fitted to data. To facilitate estimation of the parameters for both monotonic and U-shaped nonlinearities, we first fitted a single logistic nonlinearity $f\left(g\right)=b+\left(M-b\right)/\left[1+e^{-r_{o}\left(g-g_{o}\right)}\right]$ to the data, and initialized the parameters of the bi-logistic nonlinearity to describe the dominant lobe (ON or OFF, determined by the sign of $r_{o}$). Such bi-logistic nonlinearities had been previously used to describe tuning curves in sensory neuroscience under the name “difference of sigmoids” (Fischer et al., 2009; Mel et al., 2018; Murgas et al., 2020).

To assess the prediction accuracy by the LN model, we applied a normalized correlation coefficient (${CC}_{norm}$) as our model performance metric (Schoppe et al., 2016). This measure was used to account for differences in response reliability among cells, since we used a relatively small number of trials per image. Concretely, for M images and N trials per image, $R_{m,n}$ denoted the cell's response to image m for trial n, with $y_{m}=\sum_{n=1}^{N}R_{m,n}/N$ being the average response to a particular image, $\hat{y_{m}}$ being the prediction for the same image, and y and $\hat{y}$ denoting the corresponding distributions over images. ${CC}_{norm}$ was then defined as follows:

$${CC}_{norm}=\frac{Cov(y,\hat{y})}{\sqrt{Var\left(\hat{y}\right)\times SP}}$$

We calculated the necessary quantities as the sample covariance $Cov\left(y,\hat{y}\right)=\sum_{m=1}^{M}\left(y_{m}-〈y〉)(\hat{y_{m}}-〈\hat{y}〉\right)/M$ and sample variance $Var\left(\hat{y}\right)=\sum_{m=1}^{M}{\left(\hat{y_{m}}-〈\hat{y}〉\right)}^{2}/M$. *SP* denotes the signal power, a measure of signal-to-noise ratio, and is defined as follows:

$$SP=\frac{Var\left(\sum_{n=1}^{N}R_{m,n}\right)-\sum_{n=1}^{N}Var(R_{m,n})}{N(N-1)}$$

with the two variances in the numerator denoting sample variances over images.

We estimated LN model performance through 10-fold cross-validation. Briefly, the collection of average responses for all images was randomly split into 10 equally sized sets. Every set was used once as a test set for the full LN model, whose nonlinearity was fitted to the other 90% of image responses. For each cell, LN model performance was defined as the average ${CC}_{norm}$ over all cross-validation sets. For all nonlinearity visualizations in the plots, we used the nonlinearity corresponding to the cross-validation set with the ${CC}_{norm}$ value closest to the average.

Since ${CC}_{norm}$ values are ill-defined for very low data reliability, we excluded cells whose responses for identical images were highly variable. For each cell, we therefore calculated the coefficient of determination (*R*^2^) between responses averaged over even ($r_{m}^{e}$) and over odd trials ($r_{m}^{o}$), where *m* = 1,…,*M* enumerates the images. Concretely, we used a symmetrized *R*^2^, defined as follows:

$$R^{2}=1-\frac{1}{2}\times \frac{\sum_{m=1}^{M}{\left(r_{m}^{e}-r_{m}^{o}\right)}^{2}}{\sum_{m=1}^{M}{\left(r_{m}^{o}-\mu^{o}\right)}^{2}}-\frac{1}{2}\times \frac{\sum_{m=1}^{M}{\left(r_{m}^{o}-r_{m}^{e}\right)}^{2}}{\sum_{m=1}^{M}{\left(r_{m}^{e}-\mu^{e}\right)}^{2}}$$

where $\mu^{o}$ and $\mu^{e}$ are the average odd and even trial responses over images. We excluded cells with *R*^2^ < 0.5 from further analysis. We furthermore excluded cells that showed large response drift over the course of image presentations. In most cases, drift corresponded to a global scaling that approximately affected responses to all images proportionally. This is reflected in a high Pearson correlation over images between the average responses of the first five and last five trials; 94% of analyzed cells had a correlation coefficient of at least 0.7. Such global scaling of responses does not affect the analysis of differences in average responses. Thus, we excluded cells with coefficients <0.7 from further analyses. The two criteria (regarding reliability and drift) yielded 900 cells included in the analysis of 1209 recorded cells.

##### Calculation of spatial contrast (SC) sensitivity for natural images

We measured the SC of an image in the RF center of a given ganglion cell as the weighted SD of pixel contrast values inside the 2σ contour of the Gaussian center fit as follows:

$$\text{S}C=\sqrt{\frac{\sum_{i}w_{i}{\left(p_{i}-\mu_{w}\right)}^{2}}{\sum_{i}w_{i}}}$$

where the sums run over all pixels i within the 2σ contour, $p_{i}$ is the pixel value, $w_{i}$ is the pixel weight as given by the value at the pixel center of the fitted RF center part, and $\mu_{w}$ is the weighted mean of the pixel values.

To obtain the SC sensitivity, we sorted the images according to their linear predictions in the cell's LN model and then grouped neighboring images into pairs, with each image belonging to a single pair, yielding 150 pairs per cell when 300 images had been applied. For each image pair, we calculated the SC difference and the trial-averaged response difference. To compare across cells, we normalized the response differences by the maximum response (over images) of the cell. We defined the SC sensitivity as the slope of the linear regression between the SC differences and the normalized response differences. Cells were defined as contrast-sensitive if they had a significant regression slope at the 5% significance level.

##### Assessment of spatial nonlinearity with contrast-reversing gratings

To compare our findings to classical analyses of spatial integration, we stimulated the retina with full-field square-wave gratings of 100% contrast. The contrast of the gratings was reversed every 1 s. The reversing gratings were presented sequentially from higher to lower spatial frequencies for 20-30 reversals each, and the whole sequence was repeated 2 times. Depending on the experiment, we sampled 5-8 spatial frequencies, with bar widths ranging from 15 to 240 μm. For each spatial frequency, we applied 1-4 equidistant spatial phases, with more phases for lower spatial frequencies (e.g., one for 15, two for 30, two for 60, four for 120, four for 240 μm bar width). In some of the recordings, we also included contrast reversals of homogeneous illumination (corresponding to a bar width of ≥6 mm). Between presentations of the different gratings, there was a gray screen at background intensity for 2 s. We constructed peristimulus time histograms (PSTHs) over one reversal period by binning ganglion cell spikes with 10 ms bins and averaging across reversals and repeats, leaving out the first reversal after a gray period. In some experiments, gratings were flashed for 200 ms, and presentations of reversed contrast were separated by an 800 ms gray screen at background intensity. For the subsequent analyses, PSTHs corresponding to one full reversal period were constructed by extracting the cell responses during the 200 ms grating flashes and concatenating the two PSTHs for the two spatial phases of the grating into a single 400 ms PSTH. For all analyses of responses to gratings, we excluded cells with unreliable responses by calculating *R*^2^ values between average response vectors of even and odd trials, similar to the analysis of natural-image responses. We created the response vector of a single trial by concatenating single-trial PSTHs from all different spatial frequencies and phases. We only considered cells with *R*^2^ > 0.1 for our population analyses. The criterion was satisfied by 890 of 1126 cells recorded for this stimulus.

To estimate the grating spatial scale for each cell, we extracted the peak firing rate in the PSTH (across time and spatial phases) for each bar width (Krieger et al., 2017). We then fitted a logistic function (compare Natural image response predictions with a linear-nonlinear (LN) model) to the relationship of peak firing rate versus bar width, and extracted the function's midpoint as an estimate of the spatial scale. The amplitudes of harmonics of the PSTH were calculated by temporal Fourier transforms for each combination of spatial frequency and phase (Hochstein and Shapley, 1976). From the PSTHs of all spatial scales and phases, we extracted the maximum amplitude F1 at the stimulus frequency as well as the maximum amplitude F2 at twice the stimulus frequency and defined the nonlinearity index as the ratio of F2 over F1. This definition is slightly different from other approaches, where the F2/F1 ratio is calculated for each spatial scale and phase separately, with the maximum being chosen as the nonlinearity index (Hochstein and Shapley, 1976; Carcieri et al., 2003; Petrusca et al., 2007). Our approach aimed at capturing the maximum mean-luminance-induced modulation in F1 and the maximum spatial-contrast-induced modulation in F2.

##### Assessment of spatial input nonlinearities with checkerboard flashes

To assess how local visual signals are transformed in nonlinear cells, we used a stimulus that had a checkerboard layout with square tiles of either 105 or 120 µm to the side. The tiles were alternatingly assigned to two sets (A and B) so that neighboring tiles were in different sets. For each individual stimulus presentation, each set of tiles was assigned an intensity $s_{A}$ or $s_{B}$, respectively, expressed as the Weber contrast from background illumination. Similar to our presentation of natural images, these checkerboard stimuli were flashed for 200 ms with an interstimulus interval of 800 ms, during which background illumination was presented. The contrast pairs $\left(s_{A},s_{B}\right)$ were selected from a two-dimensional stimulus space, organized in polar coordinates, by using 24 equidistant angles, each with 10 equidistant radial contrast values ($\sqrt{{s_{A}}^{2}+{s_{B}}^{2}}$) between 3% and 100%, and presented in pseudorandom order. The set of all contrast pairs was presented to the retina 4 or 5 times, with a different pseudorandomly permuted sequence chosen each time. We calculated cell responses by counting the number of spikes for each ganglion cell over a 250 ms window following stimulus onset and averaging over trials. Iso-response contour lines were constructed from the cells' response profiles using MATLAB's contour function. To exclude cells with unreliable responses, we calculated *R*^2^ values across the set of all contrast combinations between spike counts averaged over even and over odd trial numbers. We only considered cells with *R*^2^ > 0.1 for our population analyses. This criterion was satisfied by 833 of 1204 cells for which the stimulus was recorded.

Rectification (RI) and convexity (CI) indices were calculated for a specific contrast level c (c=0.6 for most analyses). To quantify rectification of nonpreferred contrasts (RI), we compared the responses $r^{half}$ under stimulation with only one spatial input (e.g., $s_{A}=c$ and $s_{B}=0$, corresponding to a stimulus on one of the four half-axes of the stimulus space) to the responses $r^{oppos}$ under stimulation with this input and the other spatial input at opposite contrast ($s_{A}=c$ and $s_{B}=-c$). In cases with no direct response measurement for a particular required contrast pair, we estimated the response based on the measured responses to nearby contrast pairs, using natural neighbor interpolation, as implemented in MATLAB's scatteredInterpolant function. From all response measurements, we subtracted the background spike count, measured as the response to the (0, 0) pair, which was included as a regular stimulus in the sequence of contrast pairs. To use a single definition of RI for ON, OFF, and ON-OFF cells, we considered all four half-axes in the stimulus space (with either $s_{A}$ or $s_{B}$ at either positive or negative contrast) and computed a weighted average from the four $r^{half}$ values as well as from the corresponding $r^{oppos}$ values (there are only two $r^{oppos}$ values that are each used twice) to define RI as their ratio as follows:

$$RI=\sum_{i=1}^{4}w_{i}r_{i}^{oppos}/\sum_{i=1}^{4}w_{i}r_{i}^{half}$$

where the weights $w_{i}$ are measures of sensitivity along each half-axis i. Concretely, we obtained $w_{i}$ as the slope of a regression line, fitted to the contrast-response pairs along the corresponding half-axis.

Similarly, for quantifying integration of preferred contrasts (CI), we compared the $r^{half}$ values to responses $r^{same}$, which were measured with the same contrast for the two stimulus components, $s_{A}=s_{B}=c/2$, corresponding to the spatially homogeneous stimulus that has the same linearly integrated contrast as the stimulus used to measure $r^{half}$. Again, we took all four half-axes into account for defining CI as follows:

$$CI=1-\sum_{i=1}^{4}w_{i}r_{i}^{same}/\sum_{i=1}^{4}w_{i}r_{i}^{half}$$

We subtracted the ratio of $r^{same}$ over $r^{half}$ from unity so that CI = 0 corresponds to linear integration and CI > 0 to ${r^{same}<r}^{half}$, and thus a convex, outward-bulging shape of the iso-response contour line. We used RI and CI to formally define homogeneity-sensitive cells as cells with RI < 0 and CI < 0, corresponding to iso-response contour lines curving toward the origin.

To probe spatial integration in the RF center with minimal surround influence, we used local checkerboard flashes. The stimulus was similar to the one above, but with small patches of 2 × 2 tiles. Tiles here had a side length of 105 μm, and patches thus had a side length of 210 μm. To compare our results with the full-field version of the stimulus, patch tiles were placed to align with the tiles of the full-field stimulus. The local patches were flashed for 200 ms, with no interval between successive presentations. For each individual presentation, the applied patch locations were randomly chosen to maximally fill the screen (typical number of locations = 44-61, median = 53) while ensuring a minimum center-to-center distance of three patch side lengths (630 µm) for simultaneously presented patches (see Fig. 5*C*, bottom left). The rest of the screen was kept at background illumination (see Fig. 5*C*, top). For each presented patch, the contrast combination $\left(s_{A},s_{B}\right)$ was selected randomly and independently from the contrast combinations at other, simultaneously displayed locations. We applied fewer contrast combinations than for the full-field version of the stimulus to ensure adequate numbers of trials for each contrast combination at each location. Specifically, we used 8 or 12 equidistant angles in the stimulus space, each with 5 or 6 equidistant radial values between either 20%-100% or 3%-100%.

We also used the weights $w_{i}$ to calculate an index of relative sensitivity for the two types of tiles (A and B). For each cell, we selected the pair of half-axes (of either positive or negative $s_{A}$ and $s_{B}$ values) with the highest average weight. The relative sensitivity index was calculated as follows:

$$\frac{w_{B}-w_{A}}{\left|w_{B}\right|+\left|w_{A}\right|}$$

with $w_{A}$ and $w_{B}$ being the weights of the half-axes in the selected pair. An index of zero indicates a balanced sensitivity to both types of tiles.

For analysis, we selected for each ganglion cell the patch closest to its RF center and extracted the responses to flashes when this particular patch was used. We counted the number of spikes over a 250 ms window following presentation onset and again subtracted the background activity, which was here obtained by interpolation to the (0, 0) contrast pair. Response contour lines in stimulus space as well as rectification and convexity indices were calculated in the same way as for the full-field version of the checkerboard flashes. Similarly to the full-field stimulus, we calculated *R*^2^ values between the average spike counts of even and odd trials with respect to all contrast combinations, and only considered cells with *R*^2^ > 0.1 for our population analyses. Additionally, we required that cells had a relative sensitivity index for the Tiles A and B with an absolute value <0.5. Both criteria were satisfied by 289 of 564 cells.

##### Spatial scale estimation from blurred natural images

For recordings with blurred natural images, we selected either 30 or 40 images from our set of natural images. The images were blurred by convolution with a two-dimensional, spherically symmetric Gaussian function. We used different σ values of the Gaussian to implement different spatial scales of blurring, defined as the diameter of the 2σ Gaussian contour (Schwartz et al., 2012), to also match our RF center definition. Blurred and original images were presented in a pseudorandom sequence, similar to the presentation of the large set of natural images described above, collecting 10 trials for each image and blurring scale. Responses were again measured for each ganglion cell by counting the number of spikes over a 250 ms window following stimulus onset.

We calculated *R*^2^ values (see Natural image response predictions with a linear-nonlinear (LN) model) between blurred and original spike counts for each scale. We also calculated an *R*^2^ value for the original image responses by considering odd- and even-trial averages and assigned this value to a blurring scale of 0 μm. We then fitted logistic functions to the *R*^2^ values with respect to the blurring scales. We defined the natural spatial scale for each ganglion cell as the midpoint of the fitted logistic function. Again, by requiring *R*^2^ > 0.1 for odd- versus even-trial averages of the original images, we included 747 cells of 850 for which we had recorded the stimulus.

##### Detection of image-recurrence-sensitive (IRS) cells

We detected IRS cells as described previously (Krishnamoorthy et al., 2017). Briefly, we presented a square-wave grating of either 240 or 270 µm spatial period and 60% contrast in a sequence of 800-ms-long fixations, separated by 100 ms transitions. During a transition, the grating was shifted by approximately two spatial periods to land in one of four equidistant fixation positions (corresponding to four specific spatial phases of the grating). The sequence of the four fixation positions was randomly chosen so that all 16 possible transitions (between starting and target positions) appeared several times in the stimulus sequence.

IRS cells are described as cells that show a strong response peak after onset of the new fixation when the grating position is the same as before the transition, but not when it has reversed contrast across the transition. To detect this, as done previously (Khani and Gollisch, 2017; Krishnamoorthy et al., 2017), we measured the response after each of the 16 possible transitions by creating PSTHs with bins of 10 ms and extracting the maximal difference for successive time bins in the PSTH as a measure of response increase (maximal derivative of the PSTH) in the window from 50 to 200 ms after fixation onset. We compared for each target grating i the maximal derivative $D_{i}^{rec}$ under image recurrence (when the starting grating was also i) to the maximal derivative $D_{i}^{change}$ when the starting grating was contrast-reversed compared with grating i. We calculated a recurrence sensitivity index (RSI) as $\text{R}S\text{I}=\frac{1}{4}\sum_{i=1}^{4}\left(D_{i}^{rec}-D_{i}^{change}\right)/\left(D_{i}^{rec}+D_{i}^{change}\right)$. Cells with RSI > 0.7 and an average peak firing rate of at least 50 Hz in the post-transition PSTHs of the four image recurrences were considered as IRS cells.

##### Detection of direction-selective (DS) and orientation-selective (OS) cells

To identify DS ganglion cells, we used drifting sinusoidal gratings of 100% contrast, 240 µm spatial period, and a temporal frequency of 0.6 Hz (Sabbah et al., 2017). The gratings were shown in a sequence of eight equidistant directions with four temporal periods per direction, separated by 5 s of background illumination. The sequence was repeated 4 or 5 times. For each angle (θ), we collected the average spike responses ($r_{\theta }$) during the presentation of the grating (excluding the first period). We calculated a direction selectivity index (DSI) as the magnitude of the normalized complex sum $\sum_{\theta }r_{\theta }e^{i\theta }/\sum_{\theta }r_{\theta }$ (Mazurek et al., 2014). The preferred direction was obtained as the argument of the same sum.

We also used drifting square-wave gratings of 100% contrast, 225 µm spatial period, and a temporal frequency of 4 Hz to identify OS ganglion cells (Nath and Schwartz, 2016, 2017). The gratings were shown in a sequence of eight equidistant directions with 12 periods per direction, separated by 2 s of background illumination. The sequence was repeated 4 or 5 times. We calculated an orientation selectivity index (OSI) as the magnitude of the complex sum $\sum_{\theta }r_{\theta }e^{i2\theta }/\sum_{\theta }r_{\theta }$. The preferred orientation was obtained as the line perpendicular to half the argument of the same sum.

To calculate the statistical significance for both indices, we used a Monte Carlo permutation approach (Liu et al., 2017). For a given cell, we repeatedly shuffled the responses over all angles and trials 2000 times to obtain a distribution of DSI (or OSI) values under the null hypothesis that the firing rates are independent of the motion direction (or orientation). All cells with DSI > 0.25 (significant at 1% level) were considered as DS cells. Similarly, OS cells were identified as cells with OSI > 0.25 (significant at 1% level) that were not DS or IRS. We only included cells with a total mean firing rate >1 Hz during the presentation of the drifting gratings (Kühn and Gollisch, 2016).

OS cells were classified as either ON- or OFF-type based on the sign of the first peak (i.e., closest to zero) in the fitted temporal component $k_{T}(t)$. Here we disregarded a peak if its amplitude (unsigned) was <25% of the largest deflection.

##### Data and code availability

The spike-time data used in this study and sample code for stimulus reconstruction are available at https://gin.g-node.org/gollischlab/Karamanlis_Gollisch_2021_RGC_spiketrains_natural_image_encoding.

## Results

### Performance of LN models for predicting responses to natural images varies strongly among RGCs

**Figure 1.**
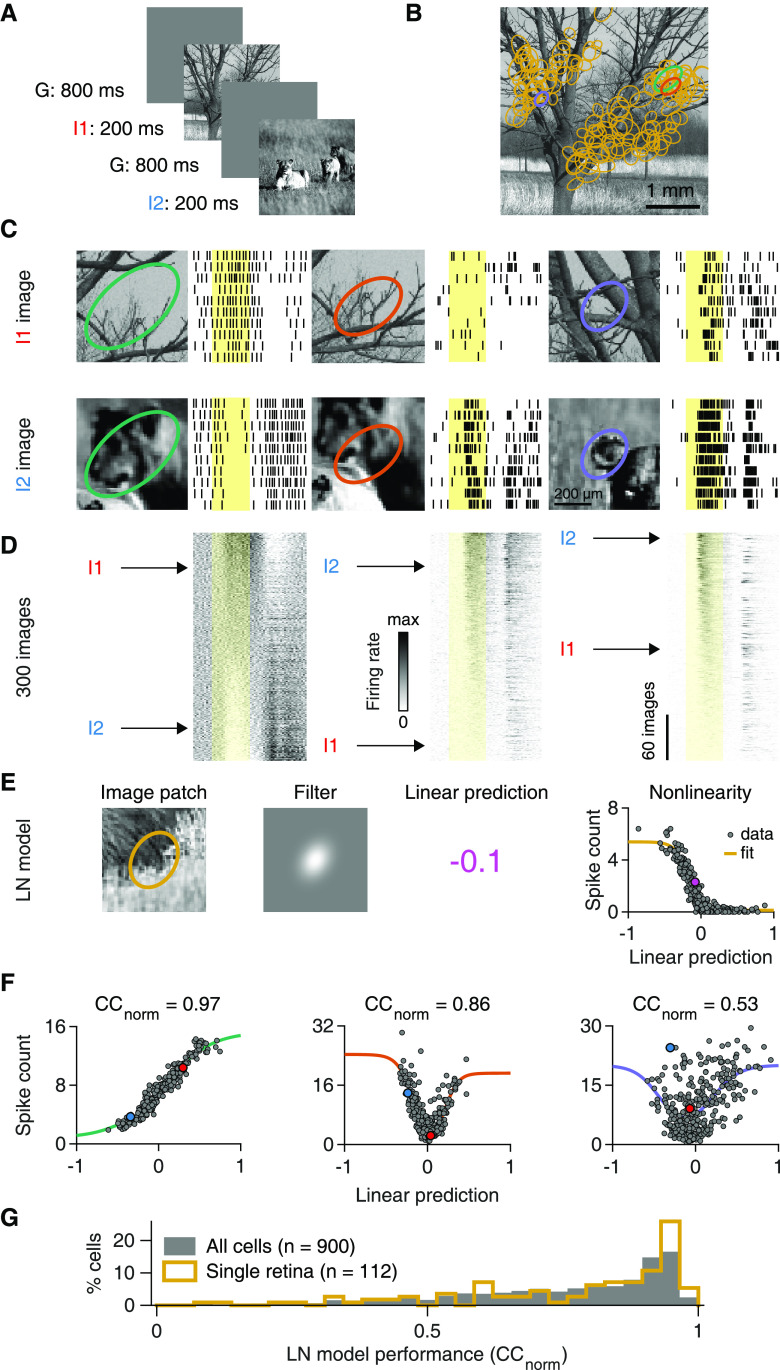
A spatially linear RF model often fails to predict natural image responses of RGCs. ***A***, Natural images were presented to the retina in a pseudorandom sequence for 200 ms with an interstimulus interval of 800 ms. ***B***, Sample natural image (I1). Overlaid ellipses (light orange) represent the outlines of RF centers (center parts of difference-of-Gaussians fits) of 130 RGCs from a single recording. The midline is RF-free because it contained the optic disk region. ***C***, Top, Raster plots with responses of three different ganglion cells to 10 presentations of image I1. Different RF outline colors correspond to different cells, also highlighted in ***B***. Bottom, Same as in top, but for presentations of another image (I2). Yellow-shaded areas correspond to the 200 ms image presentations. ***D***, PSTHs for 300 natural images, aligned to the raster plots of (***C***) and sorted by the average spike count during stimulus presentation. Rows corresponding to images I1 and I2 are marked. ***E***, The structure of an LN model that we used to predict average spike counts for natural images. The linear prediction is the inner product of the contrast values in the image patch and the filter. ***F***, Spiking nonlinearities fitted to observed spike counts for the three sample cells of ***C***. Data points for images I1 and I2 are highlighted. Top, The obtained normalized correlation coefficients (CC_norm_). ***G***, LN model performance distribution for ganglion cells in a single retina preparation (light orange, same as in ***B***), and for all recorded cells (gray) from 13 preparations (9 animals).

Since our goal was to assess the role of spatial nonlinearities in the RF, we focused on stimuli that have natural spatial structure, but simplified temporal dynamics. We therefore stimulated the retina with briefly flashed achromatic natural images while recording the spiking activity of several hundred mouse RGCs with multielectrode arrays, to survey whether linear RF models could capture the cells' responses. The images had been collected from three different databases (van Hateren and van der Schaaf, 1998; Olmos and Kingdom, 2004; Arbeláez et al., 2011) and were presented for 200 ms each, separated by 800 ms of background illumination (Fig. 1*A*). Flash duration was close to the typical fixation duration in “saccade-and-fixate” gaze patterns observed in freely moving mice (Meyer et al., 2020; Michaiel et al., 2020). To analyze ganglion cell responses in relation to the signal inside the RF, we determined the RFs (including center and surround) from responses to spatiotemporal white noise (Fig. 1*B*). Different cells sampled different parts of the images and displayed a variety of response patterns (Fig. 1*C*), with apparent sensitivity to positive or negative Weber contrast. Some ganglion cells responded to both stimulus onset and offset for some images (Fig. 1*D*), which may indicate ON-OFF-type RFs (Jacoby and Schwartz, 2017) or spatially nonlinear RFs (Mani and Schwartz, 2017). Furthermore, we observed both transient and sustained responses as well as response suppression (Fig. 1*C*, bottom left).

To test whether these diverse ganglion cell responses could originate from a spatially linear RF, we measured how well a simple linear RF model could reproduce such responses. To do so, we quantified a cell's response for each image by the average spike count over 250 ms following image onset. We then aimed at predicting this spike count with an LN model (Fig. 1*E*). The model's first stage is a linear spatial filter, which was estimated from the STA under white-noise stimulation by a parametric fit that contained a difference-of-Gaussians as the spatial component. The filter captured the location, size, shape, and relative surround contribution of the spatial RF. Applying the filter to the pixelwise Weber contrast values of a given image yielded a linear prediction: a single number that corresponded to the image's net Weber contrast as seen through the cell's RF. It quantifies how much the mean light level over the RF changed between background illumination and image presentation. The model then predicted the average spike count to the image by transforming this linear prediction with a parameterized nonlinear function, the model's nonlinearity. The nonlinearity was obtained by selecting a number of images (training set) and fitting a generic function to the relation between the linear predictions and the measured responses. The obtained nonlinearity was then used to compare predictions with actual responses for the remaining images (test set), using cross-validation to quantify prediction accuracy.

**Figure 2.**
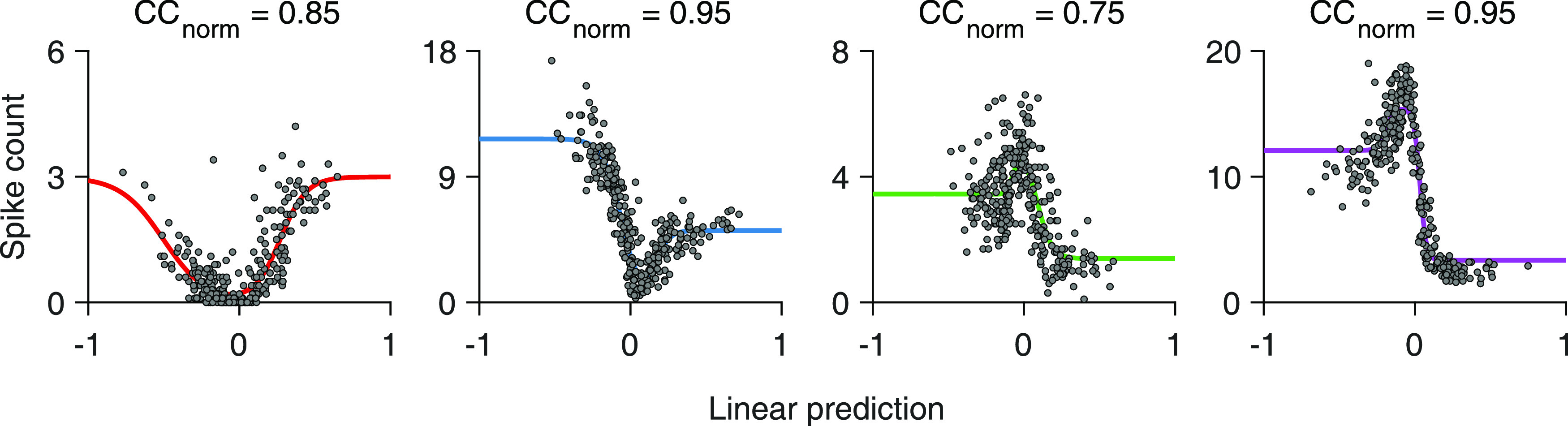
Examples of U- and bell-shaped nonlinearities. Four sample cells, with either ON-OFF-type nonlinearities (the two leftmost) or suppressed-by-contrast-type nonlinearities (the two on the right).

**Figure 3.**
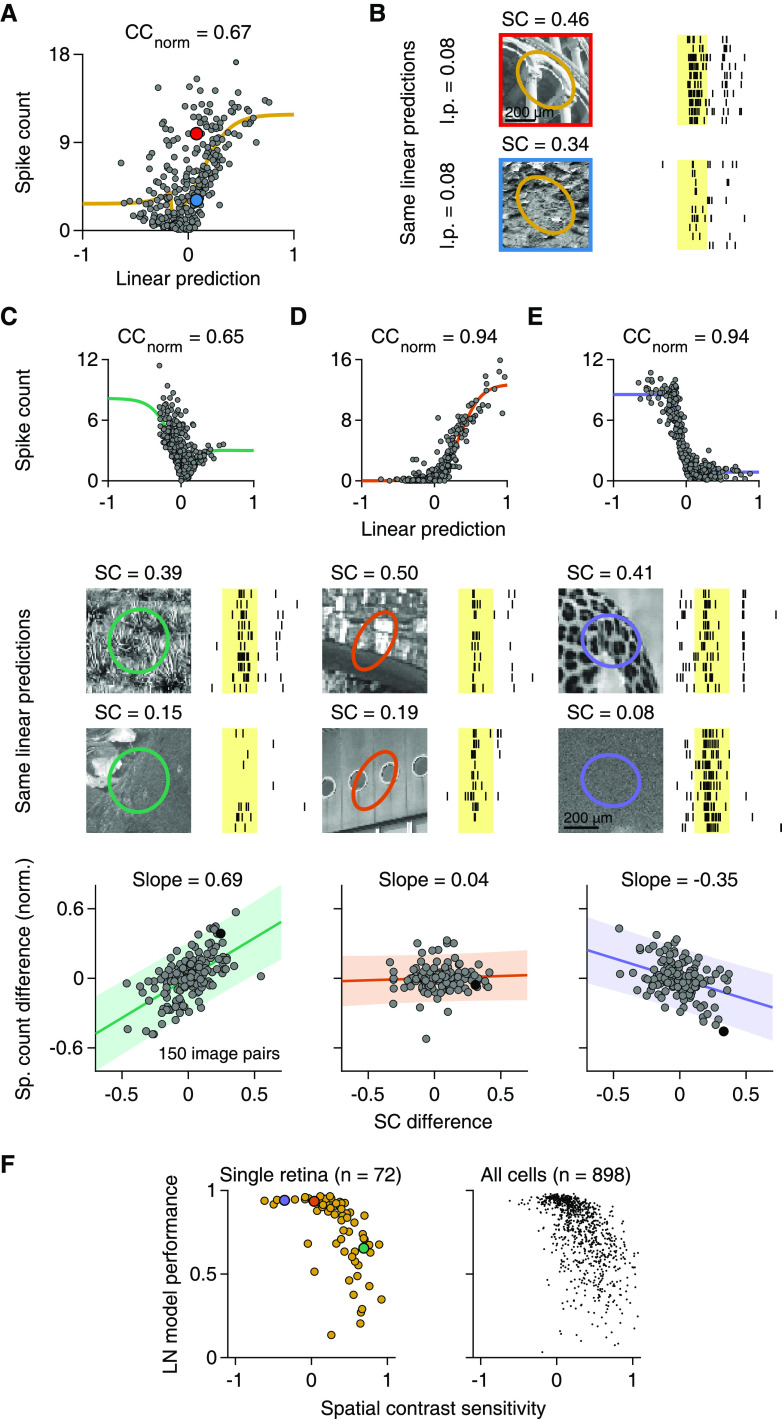
Sensitivity to natural SC is correlated with LN model performance. ***A***, Output nonlinearity fit for a cell with low LN model performance. Marked data points correspond to the responses shown in ***B***. ***B***, Different responses of the cell in ***A*** to natural images with approximately same linear predictions (l.p.), but different SC in the RF center. ***C***, Top, Output nonlinearity fit for another sample cell. Middle, Raster plots of the cell's responses to natural images with approximately same l.p., but with high (top) or low (bottom) SC in the RF center. Bottom, Relation of SC differences to average spike count differences for 150 pairs of natural images with similar l.p. in the RF center. Count differences are normalized to the maximum observed average spike count. Filled black data point represents the difference obtained from the pair of sample images above. Line and slope value correspond to least-squares estimate. Shaded area represents 95% confidence interval. ***D***, ***E***, Same as in ***C***, but for two other sample cells. ***B–E***, Shaded yellow areas represent the 200 ms image presentations. ***F***, Relation of LN model performance to SC sensitivity, defined as the slope of the relation between spike-count differences and SC differences, as in ***C–E***, for ganglion cells in a single preparation (left; Spearman's ρ = −0.73, *p* < 10^−3^) and for all recorded cells (right; 13 retinas, 9 animals). ***C–E***, Data points for the sample cells are highlighted in the corresponding colors.

For cells that linearly integrate over space, the linear prediction of the LN model should be tightly coupled to the response strength, and the relationship between the two is effectively a contrast-response function. We found cells, for example, for which the linear predictions displayed a clear, monotonic relationship to the responses, such as in Figure 1*E* (right, rank correlation between measured spike counts and corresponding model predictions: Spearman's ρ = −0.88, *n* = 300 images) or in Figure 1*F* (left, Spearman's ρ = 0.97, *n* = 300 images). As the spatial filter is defined to always have a positive central peak, an increasing monotonic relationship indicates a contrast-response function of an ON-type ganglion cell (Fig. 1*F*, left), whereas a decreasing one indicates a contrast-response function of an OFF-type ganglion cell (Fig. 1*E*). For such monotonic relationships, simple logistic nonlinearities provided good fits. Yet, we also found cells with a U-shaped relationship between linear predictions and responses (Fig. 1*F*, middle). To also capture such a nonmonotonic contrast-response function shape, we applied a bi-logistic nonlinearity, fitted to the contrast-response function of each cell. The bi-logistic functions captured nonmonotonic nonlinearities by combining an increasing and a decreasing logistic function, but also worked well for monotonic contrast-response relations, as the weight of one logistic component then naturally assumed a value near zero in the fit. Nonmonotonic contrast-response functions are expected to occur in the retina for ON-OFF (Burkhardt et al., 1998) or suppressed-by-contrast ganglion cells (Levick, 1967; Jacoby et al., 2015; Tien et al., 2015), and we indeed observed both cases as indicated by U- and bell-shaped functions, respectively (Fig. 2). The flexible parameterization of the nonlinearity allowed us to assess LN model performance and thus spatial nonlinearities for these cells in the same way as for pure ON and OFF cells. Finally, we found cells with no apparent relationship between linear predictions and responses (Fig. 1*F*, right, and Fig. 3*A*). For such cells, fits were poor because of the spread of data points, indicating that the LN model failed to predict responses to natural images.

How well the LN model captures the responses can be visually assessed by how tightly the data points cluster around the fitted nonlinearities and quantified by how strongly prediction and response are correlated. However, part of the deviation from the fit could result from noise in the response measure, as only 10 trials per image were available, rather than from an actual failure of the model. Thus, to quantify performance of the LN model, we computed a normalized correlation between response prediction and measured response, CC_norm_ (Schoppe et al., 2016), which takes the variability of responses across trials into account by assessing the model prediction relative to the reliability of the trial-averaged responses. Furthermore, we used cross-validation by averaging CC_norm_ over 10 different sets of held-out images not used to fit the nonlinearity.

Model performance varied considerably between cells. A sizeable proportion showed good model performance, indicated by a peak close to unity in the distribution of CC_norm_ values (Fig. 1*G*). On the other hand, we observed a broad tail of cells with low CC_norm_ values, indicating different degrees of model failure, both for individual retina pieces as well as for the entire population of recorded cells. Given the variability-adjusted measure of model performance and the flexibility of the applied nonlinearity, we hypothesized that the nonlinear part of the LN model was not the source of the observed diversity in natural image encoding. We therefore focused on investigating the relation between model performance and spatial signal integration.

### Linear RF model performance correlates with SC sensitivity in the RF center

Figure 3*A* displays measured spike counts versus model predictions for a sample cell with low model performance. The model failure is apparent from the fact that the cell elicited widely different spike counts for images that yielded similar linear predictions of the model, corresponding to similar net contrast over the RF, and thus similar spike count predictions. The two images shown in Figure 3*B*, for example, had nearly identical linear predictions for the sample cell, but the cell clearly responded differently to the two images. These two images strikingly differed in their spatial structures inside the cell's RF center (Fig. 3*B*). We therefore quantified the spatial structure of each image within the center of a cell's RF by computing the spatial contrast (SC, see Materials and Methods), which measures the variability of image pixels inside the RF center.

To evaluate the impact of SC on the spike output for a given cell, we grouped the images into pairs of similar linear predictions by the cell's LN model. This allowed us to relate differences in spike count within a pair to differences in SC while minimizing confounding effects of mean light-level changes inside the RF. The analysis revealed that SC was systematically related to spike count for many cells, with more spikes elicited when SC was larger (Fig. 3*C*). Indeed, for the majority of cells (72%, *n* = 651 of 898 recorded cells), differences in SC and spike count were positively correlated, indicating that SC had a response-boosting effect beyond mean light level and that spatial integration was nonlinear.

Other cells (22%, 202 of 898) appeared insensitive to SC, as indicated by an approximately flat relationship between differences in SC and spike count and no significant correlation (Fig. 3*D*). This was expected as the LN model, which is based solely on mean light level in the RF, did provide an accurate description of spike counts for some RGCs.

Unexpectedly, however, we also found a small subset of cells (5%, 45 of 898) that responded vigorously to stimuli with spatially homogeneous illumination of preferred contrast, but displayed smaller spike counts for images with similar mean illumination and higher SC (Fig. 3*E*). Such inverse sensitivity to SC represents a different form of nonlinear spatial integration than the response-boosting effect of SC in the majority of cells and may be described as sensitivity to spatially homogeneous stimulation. However, despite the inverse sensitivity to SC, the response characteristics of these cells differ from those of suppressed-by-contrast cells because the preference for homogeneous stimuli does not extend to the temporal domain. Unlike for suppressed-by-contrast cells, temporal contrast at image onset can strongly activate the cells described here (Fig. 3*E*).

To assess whether sensitivity to SC was systematically related to LN model performance, we quantified the “SC sensitivity” of a given cell by the slope of the regression line between SC and response differences, normalized by the cell's maximum response. We found that SC sensitivity was indeed negatively correlated with LN model performance in individual experiments (e.g., Fig. 3*F*, left; median Spearman's ρ = −0.60, 10 of 13 had *p* < 0.05) as well as in the pooled data (Fig. 3*F*, right; Spearman's ρ = −0.64, *p* < 10^−3^, *n* = 898 cells). Cells for which SC boosted activity (corresponding to large positive values of SC sensitivity) were generally not as well described by the LN model. This suggests that model performance is indeed limited by a systematic influence of SC on spike count in many cells. For cells with no detectable sensitivity to SC, on the other hand, model performance was generally good (CC_norm_ median = 0.91, *n* = 202). Also, the few cells with a suppressive effect of SC (negative SC sensitivity) showed fairly good LN model performance, despite the observed deviation from linear spatial integration.

### Sensitivity to fine spatial gratings alone does not predict LN model performance

**Figure 4.**
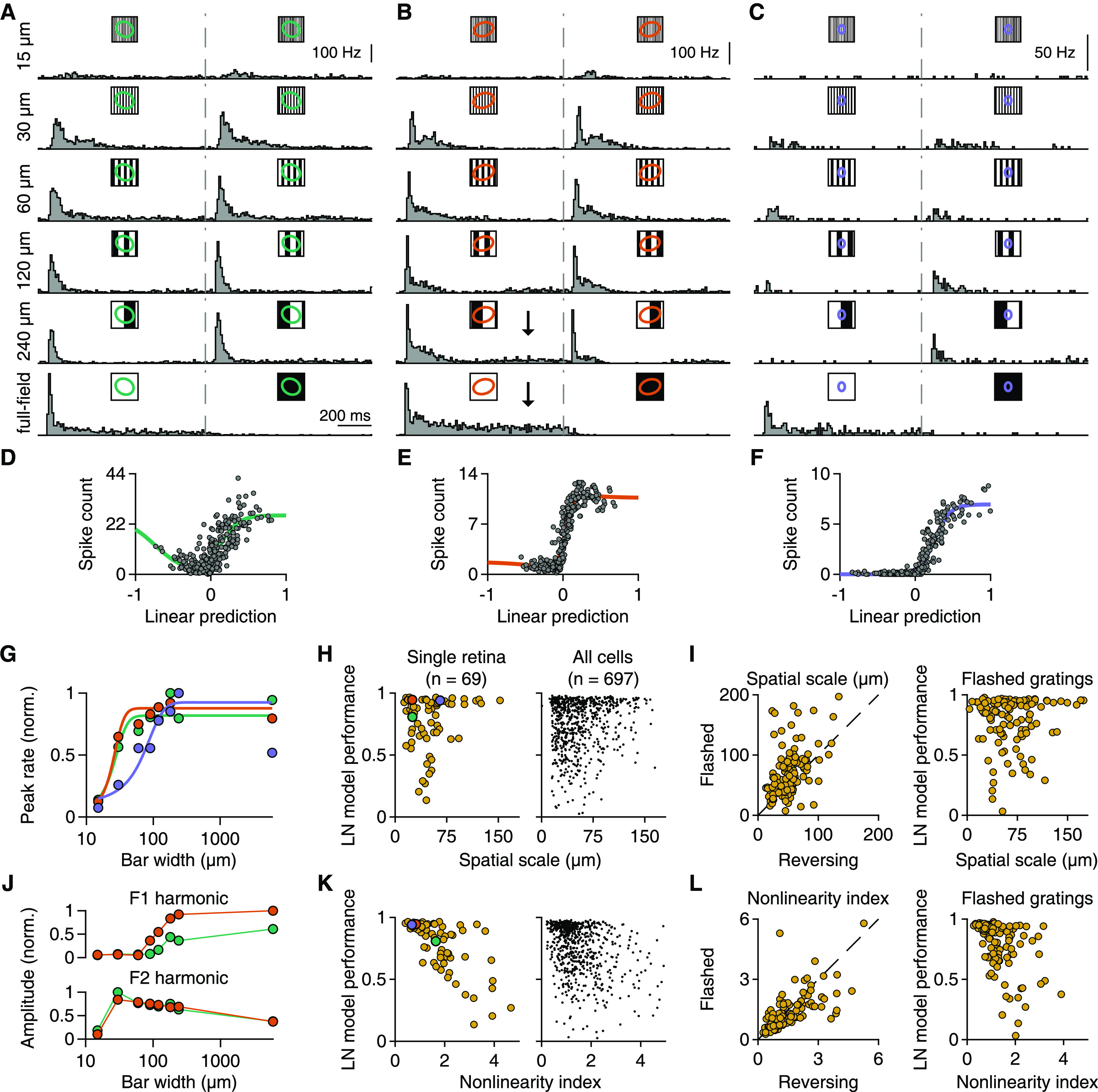
Relation of responses under reversing gratings to LN model performance. ***A–C***, PSTHs of three ganglion cells to full-field contrast-reversing gratings of six different bar widths, which are indicated to the left of the plots. For wider bars, the gratings were presented for multiple spatial phases, and the displayed PSTHs represent the phase with the smallest net brightness changes averaged over the RF. Dashed gray lines indicate the time of contrast reversal. ***B***, Arrows indicate the sustained component of the responses. ***D–F***, Relationship between linear prediction and average spike count of the same three cells for 300 natural images. Solid lines indicate fitted nonlinearities. ***G***, Relation of bar width to normalized peak firing rates (across time and spatial phases) for the cells in ***A–C***. Colors correspond to the RF colors of ***A–C***. Solid lines indicate logistic fits. ***H***, Relation of LN model performance to the spatial scale of each cell for a single retinal preparation (left, Spearman's ρ = 0.01, *p* = 0.95) and the total population (right) from 12 retinas (9 animals). Cells from ***A–C*** are highlighted. ***I***, Left, Comparison of spatial scales obtained from reversing or flashed gratings (*n* = 126). Right, Relationship between LN model performance for natural images and spatial scales from flashed gratings (*n* = 126). Data are from 3 experiments (2 animals). ***J***, Normalized F1 and F2 amplitudes (maximum over spatial phases) for the cells of ***A*** and ***B***. ***K***, Relation of LN model performance to the nonlinearity index of each cell for a single retinal preparation (left, Spearman's ρ = −0.7, *p* < 10^−3^) and the total population (right) from 12 retinas (9 animals). The nonlinearity index is defined as the maximum F2 (across spatial frequencies) over the maximum F1 (across spatial frequencies) amplitude. Cells from ***A–C*** are highlighted. ***L***, Same as in ***I***, but for nonlinearity indices.

Sensitivity to spatial structure on a sub-RF scale is characteristic for nonlinear RFs. A classical test for nonlinear spatial integration is to stimulate the retina with full-field contrast-reversing gratings at different spatial scales and phases (Hochstein and Shapley, 1976; Demb et al., 1999). Applying such stimuli in our recordings, we found RGCs that clearly responded to the reversals of fine (30 µm bar width) gratings (e.g., Fig. 4*A*,*B*), revealing nonlinear spatial integration under reversing gratings, similar to previous measurements in the mouse retina with single-cell recordings (Schwartz et al., 2012; Tien et al., 2015; Krieger et al., 2017). These cells also responded to coarser gratings that split their RF centers in two halves. Other cells, however, barely responded to reversals of gratings for phases with zero net contrast across the RF, such as the sample cell of Figure 4*C*.

Interestingly, sensitivity to contrast reversals of gratings often seemed unrelated to LN model performance for natural images. One of the sample cells with clear responses to fine-scale gratings (Fig. 4*A*) had poor LN model performance for natural images (Fig. 4*D*), whereas the other (Fig. 4*B*) showed good model performance (Fig. 4*E*). For the third sample cell (Fig. 4*C*), model performance was good (Fig. 4*F*), consistent with the observed insensitivity to grating reversals, which suggests linear spatial integration.

In order to systematically compare the sensitivity to reversals of fine gratings with the LN model performance across multiple ganglion cells, we extracted two measures from a cell's responses to the reversing gratings. First, to assess the spatial scale at which a cell becomes sensitive to the grating as revealed by a sizeable response peak (Krieger et al., 2017), we examined the cell's peak firing rates for different grating bar widths, fitted this relationship with a logistic curve, and used the curve's midpoint as a measure of spatial scale (Fig. 4*G*,*H*). Second, to assess how nonlinear spatial integration contributes to the overall strength of the response at different spatial frequencies, we compared the response Fourier components for the stimulus frequency (F1) and for twice that frequency (F2, frequency-doubled component, corresponding to responses for both reversal directions). Large F2 amplitudes, compared with F1, are indicative of nonlinear spatial-integration effects (Hochstein and Shapley, 1976) at the level of spike counts. We then computed a nonlinearity index as the ratio of the maximal F2 amplitude (over grating widths and phases) and the maximal F1 amplitude (see Materials and Methods).

This analysis showed that the spatial scale was rarely correlated to the LN model performance in individual experiments (median Spearman's ρ = 0.15, 2 of 12 experiments had *p* < 0.05; example in Fig. 4*H*, left); and for the entire dataset, this correlation was weak, albeit significant (Spearman's ρ = 0.11, *p* = 0.002, *n* = 697; Fig. 4*H*, right). Thus, sensitivity to reversals of high spatial frequency gratings, typically taken as a sign for nonlinear spatial integration, does not generally imply failure of the LN model. Indeed, many cells that start responding already for fairly fine spatial gratings (small spatial scale) showed remarkably good model performance as illustrated by the example in Figure 4*E*.

By contrast, the relative amplitudes of the F1 and F2 response components predicted model performance much better. The nonlinearity index that was computed from their ratio was negatively correlated to LN model performance under natural images both in single experiments (median Spearman's ρ = −0.37, 8 of 12 experiments had *p* < 0.05; example in Fig. 4*K*, left) as well as in the whole population (Spearman's ρ = −0.34, *p* < 10^−3^, *n* = 697; Fig. 4*K*, right). Thus, the relative degree of nonlinear spatial integration as measured by the F2 response component is a better indicator of the importance of nonlinear spatial integration under natural images than the mere sensitivity to spatial gratings.

The responses of the sample cells in Figure 4 illustrate this difference between the sensitivity to fine spatial gratings and relative size of nonlinear response components. The cells of Figure 4*A*, *B* were both sensitive already to reversing gratings of bar widths of 30 µm (Fig. 4*G*), indicative of nonlinear RFs. Yet, although initial response peaks might be similar, leading to similar F2 response components for the two cells (Fig. 4*J*, bottom), responses for the second cell were more sustained with higher spike count when net-coverage of the RF with preferred contrast was larger (see Fig. 4*B*, arrows). Thus, the responses of this cell contain also a considerable linear component even for fairly fine spatial gratings, as reflected by a higher F1 response component (Fig. 4*J*, top). The resulting lower nonlinearity index matches the better performance of the LN model for this cell. Although the linear response component may not stand out in the response patterns under reversing gratings, it may dominate the spike count responses under natural images, which contain relatively larger mean luminance signals because of the abundance of power in low spatial frequencies. Thus, even cells with clear sensitivity to fine spatial gratings and a large F2 response component under reversals may display relatively good LN model performance.

While reversing gratings are a typical stimulus used to test for spatial nonlinearities, they differ from the flashed natural images not only in their spatial structure, but also in their temporal dynamics. This might contribute to the differences observed between responses to gratings and to images. To test this, we therefore also applied flash-like presentations of gratings in some of our recordings to provide a comparable stimulation time course as for the natural images. We found that results were quite similar to those obtained with contrast-reversing gratings and led to the same conclusions. In particular, spatial scales and nonlinearity indices were correlated between the two grating versions (Spearman's ρ = 0.50, *p* < 10^−3^, for spatial scales, Fig. 4*I*, left; and ρ = 0.74, *p* < 10^−3^ for nonlinearity indices, Fig. 4*L*, left). Furthermore, similar to reversing gratings, spatial scales from flashed gratings were not informative about LN model performance, displaying no significant correlation (Spearman's ρ = 0.15, *p* = 0.11, Fig. 4*I*, right), whereas nonlinearity indices were negatively correlated to LN model performance (Spearman's ρ = −0.55, *p* < 10^−3^, Fig. 4*L*, right). This suggests that it was indeed the different spatial structure between natural images and gratings and not their temporal profiles that led to different nonlinear characteristics of some cells under these two stimulus types.

Although we found that LN model performance under natural images and the nonlinearity index from gratings are correlated, there is considerable remaining variability across cells around this relation, potentially stemming from drawbacks of the classical analysis with contrast-reversing gratings. First, for ON-OFF cells, the analysis cannot distinguish between nonlinear integration over space or over ON-type versus OFF-type inputs, as both phenomena can lead to large F2 components. Second, the analysis primarily detects that some rectification of nonpreferred contrasts exists (as effects of preferred and nonpreferred contrast do not cancel out), but is not fully determined by the degree of rectification and does not provide information about how contrast signals at different locations inside the RF are combined.

### Responses to contrast combinations inside the RF reveal the components of natural SC sensitivity

To overcome the shortcomings of classical contrast-reversing grating stimulation and explore the relationship between SC sensitivity and LN model performance more systematically, we designed a stimulus that tests a range of contrast combinations by flashing checkerboards on the retina with different light intensities for the two sets of alternating checkerboard tiles. The idea is to independently stimulate two separate sets of spatial subunits within a cell's RF with different inputs (Bölinger and Gollisch, 2012; Takeshita and Gollisch, 2014). This allows comparing responses at different contrast levels of spatially homogeneous stimulation, stimulation of only one spatial stimulus component, or stimulation with opposite contrast of the two spatial components.

**Figure 5.**
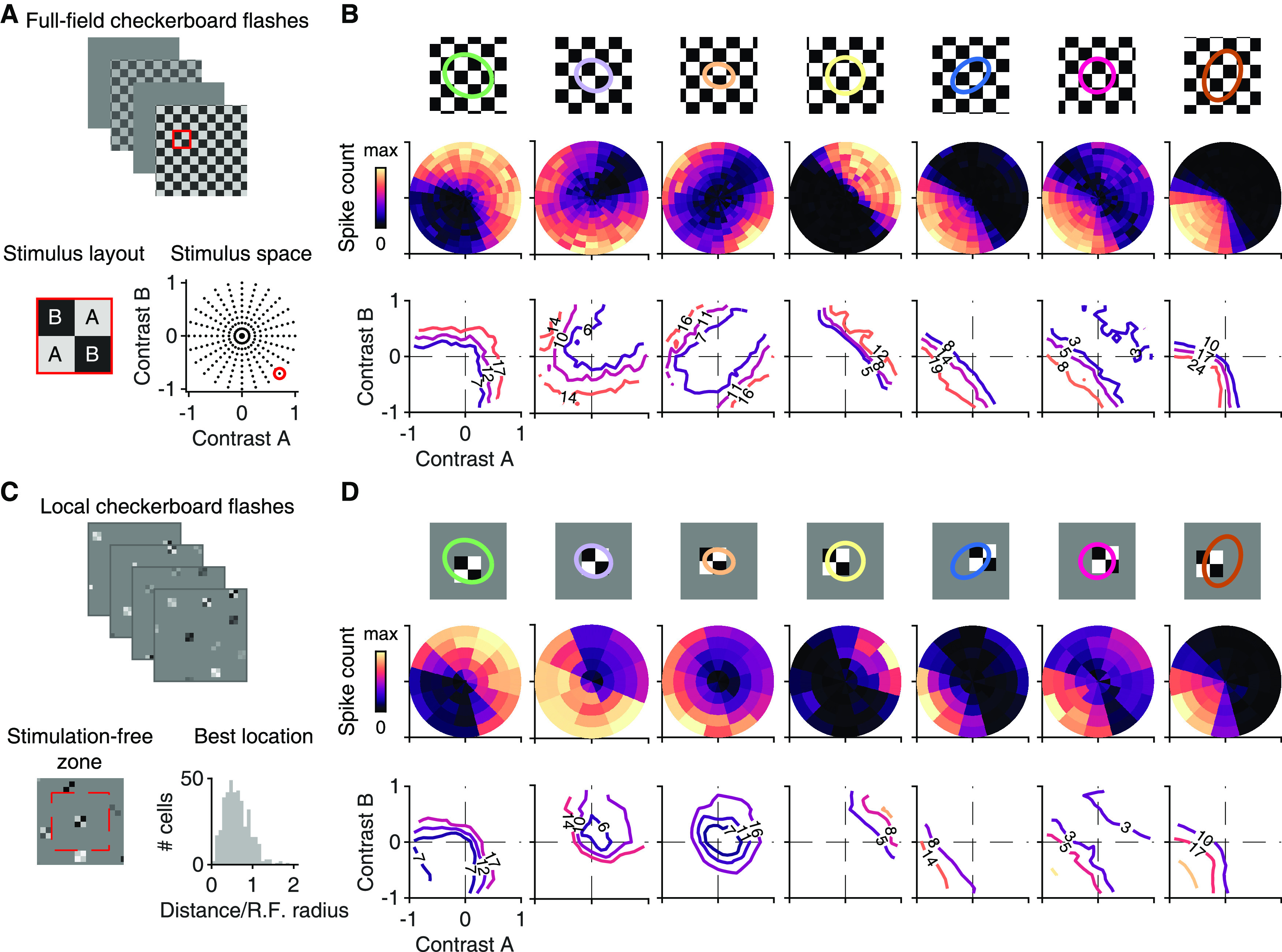
Stimulation with contrast combinations reveals nonlinearities in spatial input integration. ***A***, Depiction of the applied stimulus space, comprising flashed checkerboards with contrast combinations (A, B) sampled along different directions in stimulus space. Bottom right, Dots in the stimulus space represent all contrast combinations applied in the experiment. The contrast combination identified by the red circle corresponds to the example on the left, which shows a 2 × 2 cutout from the stimulus frame shown on top (marked by the red square). ***B***, Top, RF center outlines of seven sample ganglion cells, relative to the stimulus layout, with tiles for Contrast A and B shown in white and black, respectively. Middle, Color-coded average spike counts for all tested contrast pairs in the stimulus space. Bottom, Iso-response contours in the stimulus space for three selected spike counts (at 30%, 50%, and 70% of the maximum spike count), indicated by the number on the contour and the contour's color. Contour shapes are largely invariant to the selected response level. ***C***, Top, Example frames of the locally sparse stimulus. Bottom, Display of the region (dashed red line) that was excluded from further selection of stimulus locations around an already selected location (left). Distribution of distances from the RF midpoint to the center point of the closest grid square (*n* = 404), normalized by the RF radius. ***D***, Same as in ***B***, but for the locally sparse stimulus. Contour lines are shown for the same spike counts as in ***B***.

Concretely, we applied a batch of varied checkerboards (Fig. 5*A*, top), whose Contrasts A and B for the two sets of tiles, or spatial inputs, systematically covered the stimulus space of pairs of contrast values (Fig. 5*A*, bottom right) to explore a wide range of contrast combinations. To directly compare responses between artificial and natural stimuli, we flashed the contrast pairs for 200 ms each (the same duration as for the natural images) in a pseudorandom sequence, collecting 4 or 5 trials per pair. The subfields of the checkerboard spanned 105 or 120 μm to the side, approximately half of the average mouse RF center, to provide a strong, yet spatially structured stimulus inside the RF.

To visualize the responses for different contrast combinations, we extracted the average spike counts over 250 ms after stimulus onset, equivalent to the response measure under natural images, and displayed them as color maps over the stimulus space of contrast pairs (Fig. 5*B*, middle row). We then calculated iso-response contour lines (Fig. 5*B*, bottom row), which trace out those contrast pairs that led to the same response (here number of spikes). The shape of the iso-response contours can reveal whether stimulus integration is linear or nonlinear and is indicative of the type of subunit nonlinearity (Bölinger and Gollisch, 2012; Maheswaranathan et al., 2018). Notably, the contours are independent of any output nonlinearities that transform responses after stimulus integration has taken place, such as thresholding and saturation in the spike generation process (Gollisch and Herz, 2012). Linear integration of the two inputs, for example, leads to straight contour lines, independent of any subsequent nonlinear transformation of the summed inputs. Curved iso-response contours, on the other hand, reflect nonlinear stimulus integration, and their shape can provide information about the type of nonlinearity, as discussed below.

The iso-response analysis under flashed checkerboards revealed a variety of spatial integration profiles among different RGCs. We found both ON and OFF varieties of nonlinear cells with contour lines curving convexly around the origin (Fig. 5*B*, Cells 1 and 2). This nonlinear signature may result from an expansive transformation of local signals, such as by a threshold-quadratic function (Bölinger and Gollisch, 2012) or by a sigmoid with high threshold (Maheswaranathan et al., 2018). Quadratic integration of inputs A and B, for example, leads to circular (or elliptic) parts of the contour lines, as A^2^ + B^2^ = const is the circle equation. Furthermore, contours that run parallel to the axes in the quadrants where the stimulus components have opposite sign indicate a rectifying threshold, as one of the two input components can apparently vary without changing the response level.

We also found linear ON and OFF ganglion cells, as identified by their straight contour lines (Fig. 5*B*, Cells 4 and 5). Furthermore, our approach allowed us to visualize the spatial integration profiles of ON-OFF cells, and distinguish between spatially nonlinear and linear ON-OFF cells (Fig. 5*B*, Cells 3 and 6). Linear ON-OFF cells responded mostly to net-increases or decreases of light intensity, but not when the two contrast signals cancelled each other, leading to straight, parallel contour lines (Cell 6). On the other hand, nonlinear ON-OFF cells often had closed or nearly closed contour lines, corresponding to strong responses also for contrast combinations with opposing signs (Cell 3). Finally, we identified a unique nonlinear spatial integration profile in some cells, characterized by contour lines curving concavely away from the origin, coming closest to the origin on the diagonal of equal contrast for A and B (Fig. 5*B*, Cell 7). Such a profile indicates a particular preference to a spatially homogeneous change in light level, as a given response level can be reached with comparatively little contrast when both spatial components are stimulated in unison. We mainly found such profiles for OFF-type ganglion cells, but occasionally in ON-type cells as well (6 of 27 cells were ON-type). Cells with similar preference for homogeneous illumination of the RF have previously been observed in the salamander retina (Bölinger and Gollisch, 2012).

For comparison, we also devised a local version of checkerboard flashes to assess potential contributions of the RF surround to nonlinear spatial integration. Here, the display of each contrast combination was spatially restricted to a patch of 2 × 2 tiles of the checkerboard, which roughly corresponds to typical RF center sizes. To nonetheless cover the entire recording area and obtain sufficient sampling of contrast combinations, multiple randomly chosen patches, obeying local sparsity (Hawrylycz et al., 2016; de Vries et al., 2020), were displayed simultaneously (Fig. 5*C*), and fewer contrast combinations were sampled compared with the full-field version of the stimulus. For further analysis, we selected for each cell the patch location closest to the RF center. This generally lay not further away than one RF radius (Fig. 5*C*, bottom right), indicating good overlap of the analyzed patch location with the RF center. Furthermore, the stimulus patch did not need to fill the RF center to trigger robust responses. Also, precise centering on the RF was not required to make the two stimulus components similarly effective. If, say, a tile of Component A was closer to the RF midpoint than the other three tiles and thus more effective in influencing the response, this was approximately balanced by the second tile of Component A being further away from the midpoint than the two tiles of Component B.

Using this local version of the flashed checkerboards, we found that spatial integration profiles, as captured by the shape of the contour lines in stimulus space, were qualitatively similar under local stimulation compared with full-field stimulation (Fig. 5*D*). This indicates that it is the nonlinear stimulus integration in the RF center that determines the shape of the contour lines. As the examples show, this shape can deviate from straight lines in different ways. Rectification of nonpreferred inputs, for example, becomes visible by how the contour line bends as it progresses from the quadrant in stimulus space that corresponds to preferred contrast for both stimulus components (top right quadrant for ON cells; bottom left for OFF cells) to the two neighboring quadrants that combine positive and negative contrast. In addition, there is also nonlinear integration of preferred contrast, which is visible in a nonlinear shape of the contour line inside the quadrant that corresponds to preferred contrast of both stimulus components.

**Figure 6.**
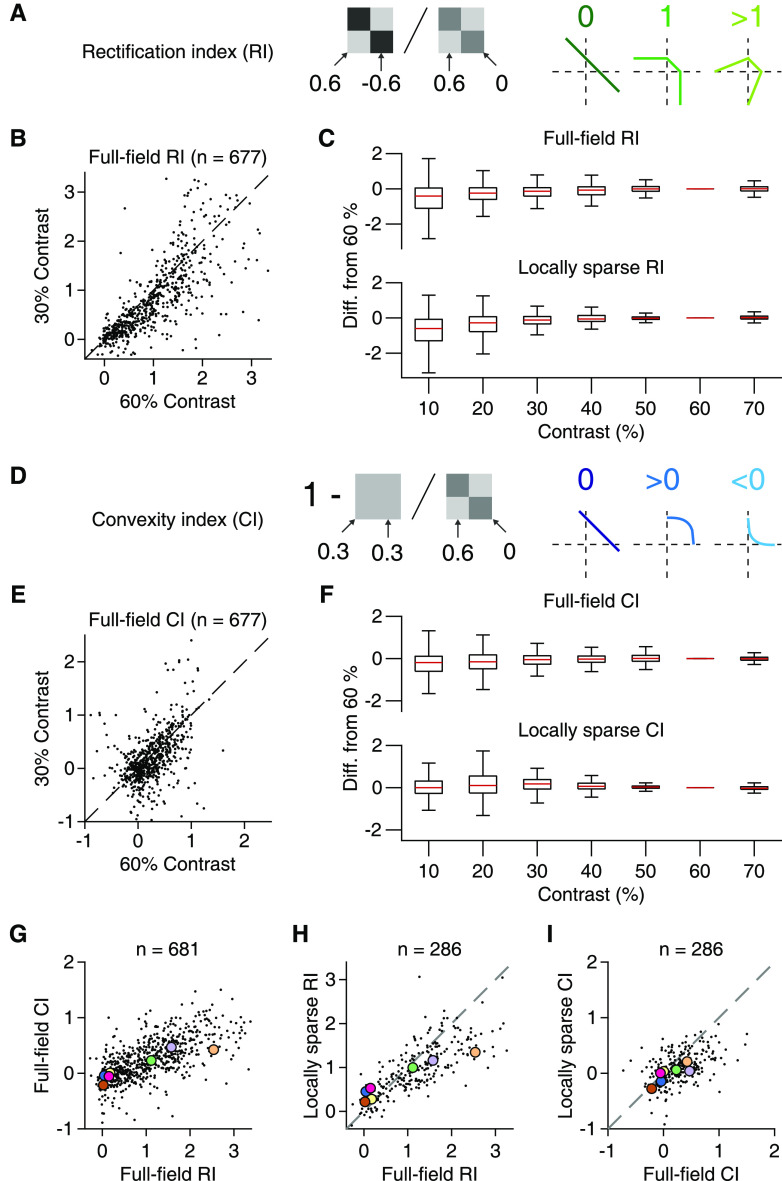
Rectification and convexity indices quantify iso-response contour shapes across contrast levels. ***A***, Schematic depiction of how rectification indices were computed from responses to different contrast combinations and how they relate to different shapes of contour lines. As indicated, the RI resulted from a ratio of responses when the tiles had opposing contrast (here 0.6 and −0.6) and when only one component was used (0.6 and 0). ***B***, Comparison of full-field rectification indices calculated for 30% and 60% contrast. ***C***, Rectification indices for different contrast levels and presentation modes (top: full-field, *n* = 695; bottom: local, *n* = 289). To compare with our default of 60%, values are shown as differences from the default. Each box represents the interquartile range (25th and 75th percentile), along with the median (red line). The whiskers extend to the maximal and minimal values, with outliers excluded (defined as data points >1.5 times the interquartile range away from the box). ***D***, Same as in ***A***, but for the CI. The CI was computed by comparing the responses when stimulation was homogeneous (here both contrast values at 0.3) and when only one component was used (contrast values 0.6 and 0) and subtracting the ratio of these responses from unity. ***E***, ***F***, Same as in ***B***, ***C***, but for the convexity indices. ***G***, Relation of full-field RI and CI in the pooled ganglion cell data from 13 retinas (9 animals). Colored dots correspond to the sample cells from Figure 5. ***H***, Relation of full-field and locally sparse rectification indices in the pooled ganglion cell data from 6 retinas (4 animals). Dashed line indicates the equality line. ***I***, Same as in ***H***, but for convexity indices.

To quantify these nonlinear signatures, we devised two corresponding indices as explained in Figure 6. We calculated a rectification index (RI, Fig. 6*A*) by comparing responses to flashes where both components had opposing, equal-magnitude contrast (here 60%; for comparison with different contrast levels, see Fig. 6*B*,*C*) with responses when only a single stimulus component was used (Molnar et al., 2009). Full rectification leads to equal responses for both configurations and an index of unity, whereas linear integration would make the opposing-contrast configuration effectively a null stimulus, resulting in no response and an index of zero. Similarly, we computed a convexity index (CI, Fig. 6*D*) by comparing responses from using just one spatial input at a specific contrast level (again 60%; compare Fig. 6*E*,*F*) with responses from using both inputs at half that contrast level. A CI of zero corresponds to linearity (equal responses for a single component at full contrast and for two components at half contrast), whereas values smaller or larger than zero correspond to increased or decreased preference for homogeneous stimuli, respectively. Over the population of all recorded cells, the two indices were correlated (for the full-field indices, see Fig. 6*G*; Spearman's ρ = 0.68, *p* < 10^−3^, *n* = 700), indicating that the two nonlinearity components often coexist and may reflect the same mechanistic origin.

To systematically compare full-field and local spatial integration profiles, we compared RI and CI across the two conditions. Although both indices displayed a significant change between local and full-field stimulation (Wilcoxon signed-rank test, *p* < 10^−3^ for both RI and CI, *n* = 289), the values were correlated between the two conditions (Spearman's ρ = 0.82, *p* < 10^−3^ for the RI and Spearman's ρ = 0.48, *p* < 10^−3^ for the CI), indicating that cells retained their relative characteristics of nonlinear spatial integration, in particular regarding rectification (Fig. 6*H*). One subtle change was that, for many cells with CI > 0 in the full-field condition, the index became smaller for the local stimulus (Fig. 6*I*), corresponding to a less outward-bulging shape of the contour line in the quadrant of preferred contrast (visible in the first two examples when comparing Fig. 5*B* and Fig. 5*D*). Thus, spatially homogeneous stimulation was less effective for these cells under full-field conditions compared with local stimulation because relatively larger contrast values were needed under homogeneous full-field stimulation to reach the activation level of the contour line. This may be explained by a linear component of spatial integration in the surround. A linear surround component would provide relatively more surround suppression under spatially homogeneous stimulation than a corresponding stimulation of only one spatial component and thereby decrease sensitivity to spatially homogeneous stimuli in the full-field condition.

**Figure 7.**
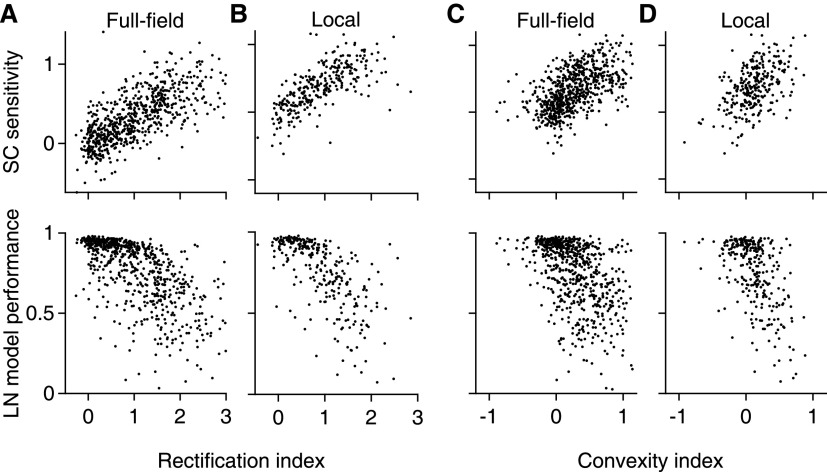
Spatial input nonlinearities correlate with SC sensitivity and model performance. ***A***, Relation of the SC sensitivity (top) and LN model performance (bottom), measured with natural images, to the full-field RI for all recorded cells (*n* = 700) from 13 retinas (9 animals). ***B***, Same as in ***A***, but using the data from the local checkerboard flash stimulus (*n* = 289) from 6 retinas (4 animals). ***C***, ***D***, Same as in ***A***, ***B***, but for the CI.

How are the extracted components of nonlinear spatial integration related to responses under natural images? We first investigated the relationship to the SC sensitivity, as determined from the responses to natural images (compare Fig. 3*C–E*), and found that it was correlated with both the RI (Spearman's ρ = 0.73, *p* < 10^−3^) and the CI (Spearman's ρ = 0.59, *p* < 10^−3^), as obtained from full-field stimulation (Fig. 7*A*,*C*, top). Similar results were also found for the indices obtained from local stimulation (Fig. 7*B*,*D*, top; Spearman's ρ = 0.75, *p* < 10^−3^ for rectification and ρ = 0.49, *p* < 10^−3^ for convexity). In line with the analysis of SC sensitivity, LN model performance for natural images also displayed a clear dependence on the rectification indices (Fig. 7*A*,*B*, bottom) from both full-field (Spearman's ρ = −0.71, *p* < 10^−3^) and local stimulation (Spearman's ρ = −0.73, *p* < 10^−3^). This relationship was much more pronounced than that between LN model performance and the nonlinearity indices extracted from contrast-reversing gratings (compare Fig. 4*K*). The convexity indices from full-field (Spearman's ρ = −0.56, *p* < 10^−3^) and local stimulation (Spearman's ρ = −0.50, *p* < 10^−3^) were also correlated to LN model performance (Fig. 7*C*,*D*, bottom), but to a smaller extent than the rectification indices. We thus concluded that the degree of rectification of spatial inputs in the RF center is a primary factor that shapes ganglion cell responses to natural images and determines whether responses can be captured by the LN model.

### The spatial scale of contrast sensitivity for natural images

**Figure 8.**
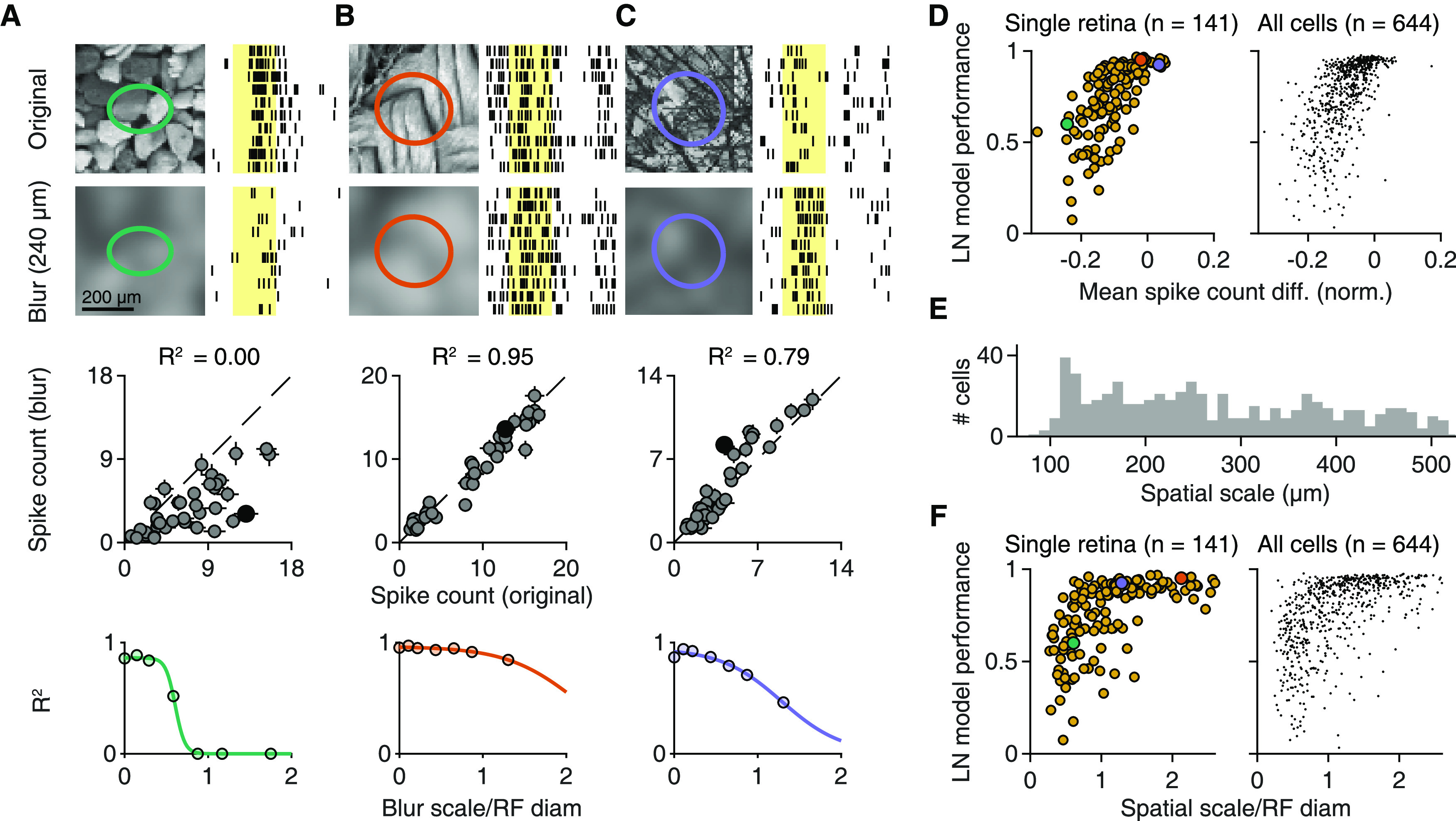
The scale of SC sensitivity for natural images. ***A–C***, Top, Responses of three sample cells to original natural images and their blurred versions for 10 trials. Images were blurred with a Gaussian function (scale 4σ = 240 μm). Shaded yellow areas represent the 200 ms image presentations. Middle, Relation of the average spike count for presentations of 40 natural images and their blurred counterparts for each of the three cells. Filled black dots correspond to the image pairs shown above. Error bars indicate SEM. Dashed line indicates the equality line. Bottom, Coefficient of determination (*R*^2^) between original and blurred spike counts for different degrees of blurring, normalized by the RF diameter of each cell. Colored lines indicate logistic fits. ***D***, Relation between LN model performance for natural images and the mean (across images) spike count difference between blurred (4σ = 240 μm) and original images for each cell from a single retina (left, Spearman's ρ = 0.75, *p* < 10^−3^) and from the pooled ganglion cell population (right, Spearman's ρ = 0.68, *p* < 10^−3^) from 9 retinas (6 animals). The differences were normalized to the maximum average spike count observed for each cell. Cells from ***A–C*** are highlighted. ***E***, The distribution of spatial scales across the pooled ganglion cell population (*n* = 644). The spatial scale was defined as the midpoint of fitted logistic functions (compare ***A–C***, bottom). ***F***, Relation of spatial scale, normalized by the RF diameter, to LN model performance for natural images for a single retina (left, Spearman's ρ = 0.63, *p* < 10^−3^) and for the pooled ganglion cell population (right, Spearman's ρ = 0.61, *p* < 10^−3^) from 9 retinas, 6 animals. Cells from ***A–C*** are highlighted.

We next asked on what spatial scale nonlinearities are relevant for encoding natural images. To do so, we compared responses under original natural images and blurred versions (Fig. 8), similar to previous analyses with white-noise patterns (Schwartz et al., 2012; Jacoby and Schwartz, 2017; Mani and Schwartz, 2017; Johnson et al., 2018). The blurring with a given spatial scale corresponds to low-pass filtering and removes fine spatial structure below this scale while keeping the mean intensity over larger regions approximately constant. At a blurring scale close to a cell's RF center diameter, blurring should diminish SC within the RF while keeping the mean light intensity approximately unchanged. Figure 8*A–C* compares responses to natural images and their blurred versions for three sample cells. At a scale of 240 µm, the blurring generally reduced responses for the first cell (Fig. 8*A*, middle; Wilcoxon signed-rank test, *n* = 40 images, *p* < 10^−3^), but left responses for the second largely unaffected (Fig. 8*B*, middle; *p* = 0.18), and for the third cell even led to increased spike count (Fig. 8*C*, middle; *p* = 0.02).

To quantify the blurring effects for all cells, we calculated the mean response difference between the blurred and the original version of the images, normalized by the cell's maximum response over all images. This spike count difference was correlated to LN model performance for natural images (Fig. 8*D*) in both individual experiments (median Spearman's ρ = 0.65, 9 of 9 experiments had *p* < 0.01), and in the pooled population (Spearman's ρ = 0.68, *p* < 10^−3^). Thus, cells that were more strongly affected by the blurring generally displayed worse LN model performance and had a stronger dependence of spike count on SC. This confirms the effect of spatial structure inside the RF for determining responses to natural images in particular ganglion cells.

When analyzing responses across different blurring scales, we observed that cells sensitive to SC reduced their spike counts already at scales smaller than their RF center (Fig. 8*A*, bottom). To quantify the spatial scale of blurring sensitivity for each cell, we measured the similarity between responses to original and blurred images by calculating the corresponding coefficient of determination (*R*^2^), which is unity when responses with and without blurring are identical, and falls off toward zero as responses to blurred images deviate more and more from the original responses. Analogous to the analysis of contrast-reversing gratings, we fitted logistic functions to the decay of *R*^2^ with blurring scale and defined the spatial scale as the midpoint of the logistic function. The obtained spatial scales ranged from 100 to 500 μm (Fig. 8*E*) and were only weakly correlated with the spatial scales measured with contrast-reversing gratings (Spearman's ρ = 0.12, *p* = 0.007). And unlike the spatial scale obtained from reversing gratings, the spatial scale from blurred images (normalized by the RF center diameter) was strongly related to LN model performance (Fig. 8*F*) in both individual experiments (median Spearman's ρ = 0.60, 9 of 9 experiments had *p* < 0.01) and in the pooled population (Spearman's ρ = 0.61, *p* < 10^−3^).

### SC sensitivity differs among RGC classes

The analyses so far have shown that the characteristics of spatial integration are consistent for individual ganglion cells across different stimulus conditions, including natural and artificial stimuli. We thus hypothesized that they reflect cell type-specific properties. To test this hypothesis, we looked at three readily identifiable cell classes, detected through a standard set of artificial stimuli.

**Figure 9.**
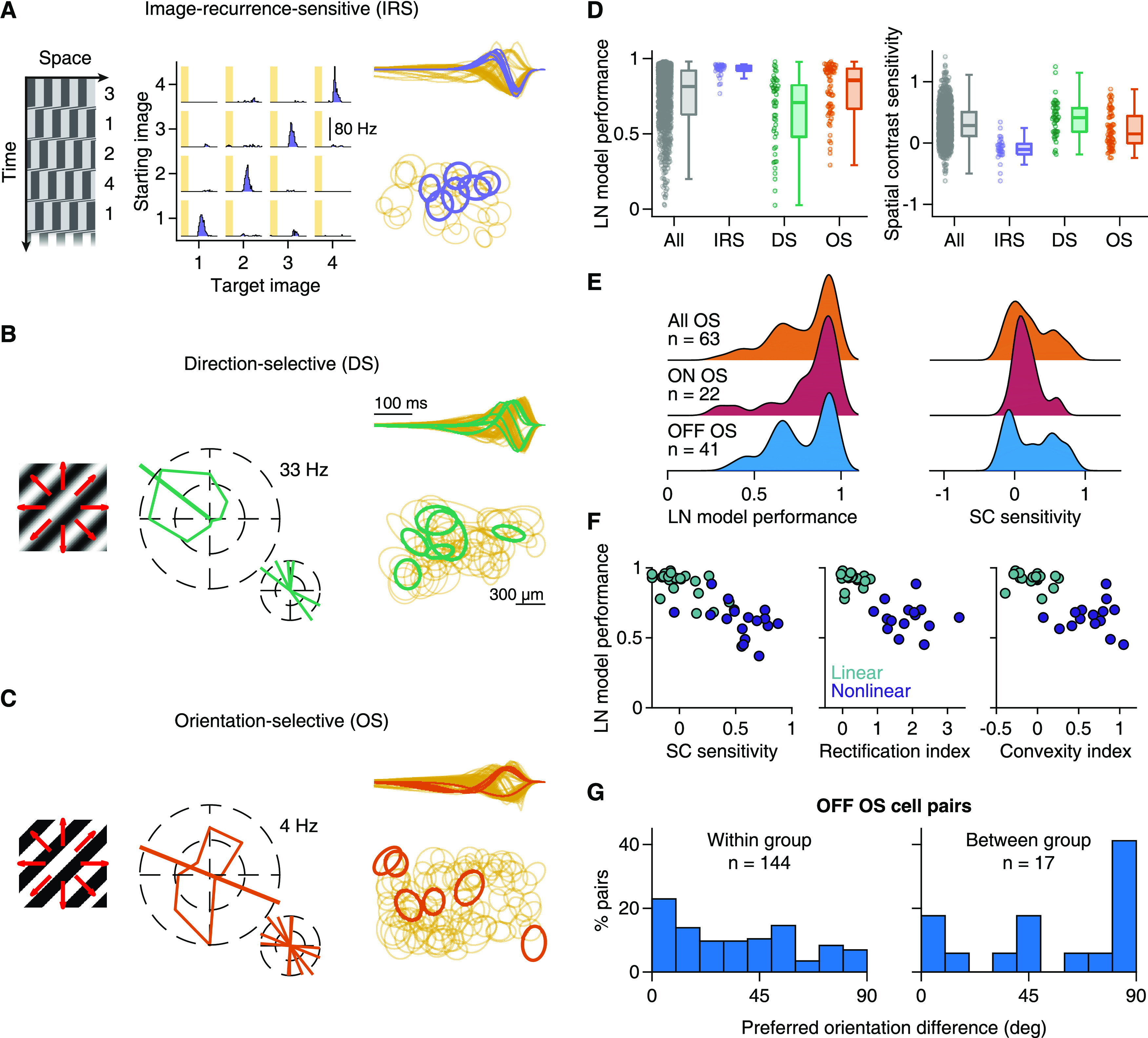
SC integration in three different functional cell classes. ***A***, IRS cells were identified with a sequence of saccadic-like grating movements (left), shown as a space-time plot with time going downward. Numbers indicate the four different fixation positions. PSTHs of a sample IRS cell for the 16 possible transitions (middle). Shaded areas represent the 100 ms transitions. Temporal filters (normalized to unit norm) of IRS cells in a single retina (right top), overlaid on top of the temporal filters of all recorded cells. Bottom, The RF centers of IRs cells (right bottom), overlaid on other nearby RFs. ***B***, DS ganglion cells were identified from their responses to slow drifting sinusoidal gratings (left). Tuning curve of a sample DS cell (middle), along with the preferred direction of all DS cells in a single recording. Temporal filters and RFs are shown as in ***A***. ***C***, OS ganglion cells were identified with drifting square-wave gratings (left), moving at higher speeds than in ***B***. Tuning curve of a sample OS cell (middle), along with the preferred orientations of all OS cells in a single recording. Temporal filters and RFs are shown as in ***A***. ***D***, Distributions of LN model performance (left) and SC sensitivity (right) under natural stimuli for IRS (*n* = 29), DS (*n* = 46), and OS (*n* = 63) cells from 13 retinas (9 animals). Gray represents all cells. ***E***, Distributions of LN model performance and SC sensitivity for ON and OFF OS cells compared with all OS ganglion cells. ***F***, OFF OS cells were assigned to two groups (linear and nonlinear) with *k*-means clustering. The features we used were the LN model performance, SC sensitivity, and full-field rectification as well as convexity indices. All four features were available for only *n* = 36 of 41 OFF OS cells, and clustering was performed with these cells only. For the other cells, responses to checkerboard flashes were not recorded, and the cells were assigned to the group whose cluster centroid was closest for the two available measures, LN model performance and SC sensitivity. ***G***, Distributions of differences in preferred orientation for pairs of OS cells that belonged to either the same group (“within group”) or to different groups (“between group”).

First, we focused on IRS cells, which form a single functional cell type in the mouse retina and which correspond to transient OFF-α ganglion cells (Krishnamoorthy et al., 2017). We identified IRS cells by their characteristic response peaks to rapid shifts of a grating with no net displacement of the grating position (Fig. 9*A*). As expected, IRS cells were all OFF-type, with fast temporal filters and tiling RFs. For these cells, all our spatial integration measures displayed relatively narrow distributions. LN model performance for IRS cells was high (Fig. 9*D*, left; median = 0.94, *n* = 29), suggesting linear spatial integration. However, rather than showing no sensitivity to SC, the distribution of SC sensitivity for IRS cells was significantly shifted toward negative values (Fig. 9*D*, right; median = −0.11, *n* = 29, Wilcoxon sign-rank test, *p* = 0.005). This indicates that IRS cells had a particular preference for spatially homogeneous natural stimuli. Specifically, about half (14 of 29) of the IRS cells were inversely sensitive to SC of natural images, as identified by a significant negative slope comparing differences in SC and in spike count for image pairs with similar mean illumination (compare Fig. 3*C–E*). In terms of spatial integration measured by the checkerboard flashes, most IRS cells showed profiles, such as the one in Figure 5*B* (Cell 7), with low rectification (median = 0.12, *n* = 28) and slightly negative convexity indices, yet not significantly different from zero (median = −0.06, Wilcoxon sign-rank test, *p* = 0.07).

Second, we tested DS ganglion cells (Fig. 9*B*), detected through their responses to drifting gratings. DS cells had either ON- or OFF-type temporal filters (Fig. 9*B*, right top), with OFF-type filters likely corresponding to ON-OFF DS cells. DS cells with OFF-type filters typically showed U-shaped nonlinearities in LN models obtained from white-noise stimulation (data not shown) and responses under light-intensity steps to both increasing and decreasing intensity. DS cells generally displayed rather nonlinear spatial integration for natural images (Fig. 9*D*), with low LN model performance (median = 0.71, *n* = 46) and significant SC sensitivity (median = 0.41, *n* = 46, Wilcoxon sign-rank test, *p* < 10^−3^). As expected for cells sensitive to SC, their full-field rectification indices were rather large (median = 1.46, *n* = 31), and their convexity indices were significantly larger than zero (median = 0.25, *n* = 31, Wilcoxon sign-rank test, *p* < 10^−3^). Thus, DS cells showed nonlinear spatial integration under both natural and artificial stimuli.

Finally, using drifting gratings with higher speed, we identified OS ganglion cells (Fig. 9*C*). We found both ON- and OFF-type OS cells, possibly corresponding to the recently described different classes in the mouse retina (Nath and Schwartz, 2016, 2017). OS cells displayed characteristics of spatial integration that lay in between IRS and DS cells (Fig. 9*D*). Compared with DS cells, for example, OS cells showed better LN model performance (Wilcoxon rank-sum test, *p* < 10^−3^), yet many cells still revealed poor model predictions (median = 0.85, *n* = 63). Likewise, SC sensitivity was lower than for DS cells, yet still significantly larger than zero (median = 0.15, *n* = 63, Wilcoxon sign-rank test, *p* < 10^−3^). This was also reflected in the OS cells' responses to the checkerboard flashes, with lower full-field rectification (median = 0.46, *n* = 50) and convexity indices (median = 0.13, *n* = 50) compared with DS cells, yet with both indices significantly larger than zero (Wilcoxon sign-rank test, *p* < 10^−3^ for rectification and *p* = 0.005 for convexity indices).

Examining the distributions of these measures for OS cells more closely, we observed that they appeared to be bimodal (Fig. 9*E*), which may indicate that different types of OS cells differ in how nonlinear their spatial integration is. Indeed, we found that ON-type OS cells showed fairly linear spatial integration characteristics (Fig. 9*E*), whereas OFF-type OS cells could be clustered into two separate groups: one with linear spatial integration and good LN model performance and another with nonlinear spatial integration and poorer LN model performance (Fig. 9*F*). Interestingly, the linear and nonlinear OFF OS cells also differed systematically in their preferred orientations (Wilcoxon rank-sum test, *p* = 0.006; Fig. 9*G*); cells from different clusters (i.e., one linear and one nonlinear cell) often had orthogonal preferred orientations, whereas cells from the same cluster more often also had similar preferred orientations. Although we do not know the preferred orientations of these cells in absolute retinal coordinates, our data suggest that linear and nonlinear OFF-type OS cells have different preferred orientations and might correspond to the previously described classes of OS cells with preference for horizontal or vertical orientations (Nath and Schwartz, 2016, 2017).

## Discussion

In this work, we directly addressed the question whether mouse RGC responses to natural images are consistent with a linear RF. This was the case only for a subset of cells (Fig. 1), as SC inside the RF influenced responses for many cells beyond the mean stimulus intensity (Fig. 3). Interestingly, classical identification of sensitivity to contrast-reversing high-frequency gratings provided only a moderate prediction of which cells are affected by RF nonlinearities under natural images (Fig. 4). We therefore devised a new stimulus to characterize subunit nonlinearities in detail for many simultaneously recorded cells, revealing considerable variability in the characteristics of nonlinear stimulus integration (Figs. 5, 6) and providing a better prediction of the relevance of RF nonlinearities for natural images (Fig. 7). Experiments with blurred natural images corroborated that nonlinear RFs affect responses under natural images and that specific ganglion cells are inversely sensitive to SC (Fig. 8). Finally, the relevance of nonlinear RFs appears to be cell type-specific and may help differentiate subtypes within broader functional cell classes (Fig. 9).

### Diversity in natural stimulus encoding among the retina's output channels

Using a simple linear RF model, we observed multiple facets of natural image encoding in the mouse retina. We found ganglion cells that were consistent and others that were inconsistent, to different extents, with linear RFs. Of the few previous studies with natural stimuli in the mouse retina, one supports generally linear RFs in mouse ganglion cells (Nirenberg and Pandarinath, 2012). However, both spatially linear and nonlinear ganglion cell types had been identified in the mouse retina with artificial stimuli. For example, the PixON (Johnson et al., 2018) or the sustained OFF-α cells (Krieger et al., 2017) appear to have linear RFs, whereas nonlinear RF properties can be detected for ON-delayed or ON-OFF DS cells (Mani and Schwartz, 2017). Here, we showed that mouse DS cells, like several other ganglion cells, are spatially nonlinear also for natural images.

Related investigations with natural images in other species have shown, for example, that the macaque retina also contains cells with linear as well as nonlinear RFs (Turner and Rieke, 2016). Our work demonstrates that this also holds for mouse retina where, moreover, we identify a surprising diversity of spatial integration, including linear and nonlinear cells within broader cell classes (e.g., within ON-OFF or OS cells) as well as cells that are inversely sensitive to SC. In rabbit retina, ganglion cell responses to natural images had also been found to deviate from linear RFs in different ways (Cao et al., 2011). Similar to our approach, this study used the dependence of image-evoked responses on the local variability of pixel intensities beyond the mean intensity as a signature of nonlinear spatial integration under natural images. Cao et al. (2011) then went on to demonstrate that the skewed distribution of intensity values in natural scenes affects ganglion cell responses via this texture sensitivity, whereas our work here focuses on characterizing the features and variability of spatial nonlinearities across ganglion cells.

The degree and type of the spatial nonlinearity appear to differ between RGC types, yet functional cell-type classification schemes rely mostly on linear model components, such as RF size and temporal filter shapes (Chichilnisky and Kalmar, 2002; Baden et al., 2016; Franke et al., 2017; Jouty et al., 2018; Ravi et al., 2018; Rhoades et al., 2019). Including characteristics of nonlinear spatial integration, such as LN model performance, subunit rectification, or spatial scale of nonlinear integration, may help to better distinguish cell types. For example, the mouse retina contains at least four subtypes of OS cells, two of which (ON- and OFF-type) are tuned to horizontal and the other two (again ON and OFF) to vertical orientations (Nath and Schwartz, 2016, 2017). Here, we found distinct groups of OS cells with linear and nonlinear spatial integration, suggesting that OS subtypes might differ not only in contrast preference or preferred orientation, but also in how they integrate SC, which provides additional information for separating and identifying subtypes of OS cells.

When considering that synaptic transmission is often inherently nonlinear, the occurrence of linear RFs may actually be surprising (Shapley, 2009). In the salamander retina, for example, nonlinear ganglion cell RF centers and surrounds seem to be the norm (Bölinger and Gollisch, 2012; Takeshita and Gollisch, 2014). Linear RFs may be a property of mammalian retinas, as they have been described also in cat, rabbit, and macaque retinas (Enroth-Cugell and Robson, 1966; White et al., 2002; Petrusca et al., 2007; Molnar et al., 2009), and may have specifically evolved to provide raw information about illumination patterns to the cortex for further processing (Roska and Meister, 2014).

### A cell class with particular sensitivity to spatial homogeneity of natural images

We identified cells in the mouse retina with particular sensitivity to spatially homogeneous regions in the images. Specifically, these cells were inversely sensitive to SC: although well described by an LN model, they respond more strongly to homogeneous stimuli than to structured stimuli of equal mean light level. This feature is not to be confused with the characteristics of suppressed-by-contrast cells (Levick, 1967; Tien et al., 2015; Jacoby and Schwartz, 2018), which are also known as uniformity detectors, and which are suppressed below baseline activity by (temporal) contrast. The homogeneity-preferring cells identified here, on the other hand, are generally activated by a new image and particularly strongly so if a spatially homogeneous region of preferred contrast falls onto the RF. This is reminiscent of the homogeneity detectors that have been described in the salamander retina (Bölinger and Gollisch, 2012), although the latter showed rectification of nonpreferred contrasts, unlike the homogeneity-sensitive cells described here. These cells, through their particular sensitivity to homogeneous stimuli, could provide information about image focus; blurring through defocusing will increase activity for this cell type and simultaneously decrease activity for spatial-contrast-sensitive cells, such as ON-delayed cells (Mani and Schwartz, 2017), which have been implicated in focus-sensing functions. A readout based on activity differences between cells of opposite tuning under image blur could provide a code for image focus that is particularly robust, for example, to variations in contrast and spatial structure (Kühn and Gollisch, 2019).

IRS cells appear to be part of the homogeneity-sensitive cells. The IRS cells correspond to transient OFF-α cells (Krishnamoorthy et al., 2017) and should therefore also match the PV5 ganglion cells, which have been shown to be approach-sensitive (Münch et al., 2009). It seems that approach sensitivity, image-recurrence sensitivity, and sensitivity to homogeneous natural images may rely on the same circuit component: strong, local (glycinergic) ON-type inhibition, which transient OFF-α cells are known to receive (van Wyk et al., 2009), and which needs to be suppressed, perhaps below baseline level, by OFF-type stimuli for maximal activity.

### Assessing nonlinear spatial integration with artificial and natural stimuli

The classical test for nonlinear spatial integration in the retina is to check for frequency-doubled responses under contrast-reversing spatial gratings whose spatial frequency is below the resolution of the linear RF (Hochstein and Shapley, 1976; Krieger et al., 2017). Yet, we found that sensitivity to fine gratings is generally not a good predictor for relevant spatial nonlinearities under natural stimuli, as measured by a failure of the LN model (Fig. 4). Several aspects likely contribute to this discrepancy. Perhaps most importantly, fine gratings isolate responses to high spatial frequencies and therefore sensitively detect nonlinear response components. Natural stimuli, on the other hand, have a broad frequency spectrum, and linear responses to the prevalent low frequencies may dominate the responses even when reversing gratings reveal nonlinear spatial integration. In addition, analyses under reversing gratings can be confounded by sensitivity to both light increments and decrements in ON-OFF cells. Finally, the high contrast typically used with reversing gratings may emphasize nonlinear effects, since higher contrast makes nonlinearities more pronounced (Turner and Rieke, 2016). The difference in temporal structure between reversing gratings and flashed images, on the other hand, did not seem to play a major role (Fig. 4*I*,*L*).

### Mechanisms of linear and nonlinear spatial integration

Nonlinear spatial integration as measured with gratings is attributed to the rectified excitation that bipolar cells provide to the ganglion cell (Demb et al., 2001). The same mechanism likely also dominates the nonlinear response characteristics under natural images, as underscored by the relation between signal rectification and LN model failure (Fig. 7). Biophysically, rectification of bipolar cell signals seems to originate presynaptically from a nonlinear dependence of vesicle release on calcium concentration in the synaptic terminal (Singer and Diamond, 2003; Jarsky et al., 2011). The nonlinear integration of preferred contrast signals, which we quantified in the CI, may have a similar origin, as vesicle exocytosis and postsynaptic currents increase supralinearly with increasing calcium concentration, at least for moderate levels (Jarsky et al., 2011).

Yet, the degree of nonlinear spatial integration varied widely across cells, suggesting different levels of partial rectification in the signal transmission from bipolar to ganglion cells. What is the origin of this variability in the degree of rectification among ganglion cell types? Presynaptically, baseline activity of bipolar cell synapses may vary, allowing some synapses to modulate transmitter release in both directions and precluding others from decreasing activity much below baseline, thus causing rectified transmitter release. For example, regarding inputs to Y-type ganglion cells in guinea pig and mouse, the basal glutamate release is higher at the more linear ON (compared with OFF) bipolar cell terminals (Zaghloul et al., 2003; Borghuis et al., 2013). Further mechanisms, such as postsynaptic receptor dynamics and inhibition, may also contribute in shaping signal transmission between bipolar and ganglion cells. Crossover inhibition from glycinergic amacrine cells (Werblin, 2010), for example, can provide response suppression below baseline for nonpreferred contrast and thereby (partially) linearize the rectified pure bipolar cell signals. The crossover inhibition can act on bipolar cell terminals (Molnar et al., 2009) or directly on the ganglion cell, and its gain, relative to the gain of the preferred-contrast excitation, would determine the degree of nonlinearity in spatial integration.

The spatial scale of nonlinear spatial integration that we identified through the presentation of blurred images (100-120 µm; Fig. 8) is somewhat larger than typical bipolar cell RFs in mouse retina of ∼40-60 µm (Berntson and Taylor, 2000; Schwartz et al., 2012; Franke et al., 2017). This might be an effect of the spatial correlations in natural images, which reduce the impact of blurring. Furthermore, electrical coupling between bipolar cells may increase the spatial scale, especially for stimuli with considerable spatiotemporal correlations (Kuo et al., 2016).

### Limitations of this study

The lack of anatomic or genetic information often complicates the clear identification of individual ganglion cell types in extracellular multielectrode array recordings. On the other hand, these high-throughput recordings can provide an overview that highlights the diversity of response properties in a way not easily possible with targeted single-cell recordings. Another issue with multielectrode array recordings is the distribution of recorded ganglion cell RFs over the broad range of the recording sites. Under natural images, different cells are stimulated by different image regions, which contributes variability among cells of the same type, as some cells may experience more spatial structure within the presented images than others. The lack of RF information may also present a problem for artificial stimuli that should target, for example, the RF center. However, our application of locally sparse stimulus presentations shows that this can be overcome, allowing high-throughput investigations of center-surround effects (Figs. 5–7), as previously used in single-cell patch-clamp recordings (Turner et al., 2018).

To analyze the cells' sensitivity to spatial structure beyond mean light intensity, we analyzed the effect of SC within the RF center, defined via the variance of pixel intensities. This simple and straightforward quantification of spatial structure fails to capture which aspects of natural images provide the most relevant SC, which may result, for example, from object boundaries, textures, or gradients in light intensity. Once sensitivity to SC is established, follow-up investigations may ask which of these natural image features might be most relevant for mediating the SC effects.

We used flashed image presentations because our study was focused on spatial integration. Thus, while the applied stimuli had natural spatial structure, they lacked, for example, motion components that are induced by eye movements. This simplification of actual natural stimuli allowed us to specifically target spatial nonlinearities without having to explicitly consider the influence of temporal filtering and adaptation on the responses. It seems likely that nonlinear spatial integration observed under our flashed natural images will also shape responses to natural movies. On the other hand, our approach is insensitive to nonlinearities triggered through specific temporal stimulus features. For example, IRS cells, which we here reported as being rather linear for the encoding of images flashed in isolation, can reveal nonlinearities when rapid image transitions are considered, for which disinhibitory interactions mediate a sensitivity to recurring spatial patterns (Krishnamoorthy et al., 2017). Additionally, we focused on a single light level, but spatial nonlinearities may change with light level: sustained ON-α ganglion cells in the mouse retina, for example, become more linear with decreasing light intensity (Grimes et al., 2014).

The presentation of full-field natural images stimulates both the RF center and surround. Our modeling approach aimed at capturing effects of surround suppression by using a difference of Gaussians as a spatial filter, which could contain positive and negative values. Yet, this might not reflect the actual surround strength under natural images because the surround might be underestimated with spatiotemporal white noise (Wienbar and Schwartz, 2018) and because the extraction of the spatial filter from the spatiotemporal STA, which often lacks space-time separability (Cowan et al., 2016), may further diminish the surround component. We thus cannot exclude that disregarded surround effects contribute to shortcomings of the LN model. However, the following two arguments indicate that, regardless of surround effects, nonlinear integration in the RF center is a main factor in LN model performance. First, our SC sensitivity analysis showed that across-cell differences in LN model performance could be explained to a large degree by considering SC only in the RF center. And second, rectification indices obtained from the full-field and from the locally restricted checkerboard flashes worked about equally well to explain the performance differences of the LN model.

More generally, the good correspondence between measures of spatial nonlinearity and LN model performance, in particular the fact that cells with little SC sensitivity (Fig. 3) or rectification (Fig. 7) displayed model performance near unity, supports the suitability of our approach to apply a parameterized LN model with fitted spatial filters and nonlinearities for assessing spatial nonlinearities in the encoding of natural images. It also underscores the reliability of the recorded data, reaffirming that observed variability in a cell's response to different images results from the cell's differential activation by the images and not from drift or rundown over the course of the long *in vitro* recordings.

### Implications for neuronal modeling

Proposed improvements to LN-type models go in many directions (Latimer et al., 2019; Shi et al., 2019). Here we demonstrated that the incorporation of sensitivity to fine spatial structure into models (e.g., with spatial subunits) should be significant for natural stimuli. We found that cells with low LN model performance mostly showed nonlinear spatial integration and that rectification of nonpreferred contrast in the RF center was particularly important. This observation of the importance of rectification agrees with results from nonlinear subunit modeling of ganglion cells: in the macaque retina, the rectification of subunits determines the degree of nonlinear integration under natural images (Turner and Rieke, 2016); and in a model of salamander RGCs under white-noise stimulation, threshold-linear rectification of subunit signals worked nearly as well as more elaborate, fitted shapes (Real et al., 2017). However, we here also found that there is considerable variability in the type of subunit nonlinearities with different degrees of rectification and convexity as well as cells with inverse sensitivity to SC. This suggests that not all cells will be well captured by a standard subunit model with summation over half-wave rectified local signals. Our approach of analyzing SC and iso-response stimuli rather than assessing the performance of an explicit subunit model allowed us to capture this diversity. Furthermore, the checkerboard flash stimulation introduced here can be used to efficiently estimate the characteristics of subunit nonlinearities for many RGCs simultaneously. Given the recently developed techniques for estimating subunit locations (Liu et al., 2017; Maheswaranathan et al., 2018; Shah et al., 2020), this paves the way for building more detailed models for different ganglion cell types. Our results also indicate that such cell type-specific approaches may be needed as there might not be a satisfactory single “standard model” (Carandini et al., 2005).
